# A Phenazine‐Based Two‐Dimensional Covalent Organic Framework for Photochemical CO_2_ Reduction with Increased Selectivity for Two‐Carbon Products

**DOI:** 10.1002/anie.202502799

**Published:** 2025-03-18

**Authors:** Zoheb Hirani, Neil M. Schweitzer, Edon Vitaku, William R. Dichtel

**Affiliations:** ^1^ Department of Chemistry Northwestern University 2145 Sheridan Road Evanston IL 60208 USA; ^2^ Department of Chemical and Biological Engineering Northwestern University 2145 Sheridan Road Evanston IL 60208 USA

**Keywords:** Carbon dioxide, Covalent organic frameworks, Organic semiconductors, Photocatalysis, 2D polymers

## Abstract

The reduction of carbon dioxide (CO₂) into valuable products will contribute to sustainable carbon use. Here we report the photocatalytic reduction of CO₂ to carbon monoxide, formate, and oxalate ions using a redox‐active phenazine‐based 2D covalent organic framework (**Phen‐COF**) and its phenazine monomer. Under similar irradiation conditions, **Phen‐COF** produced 2.9 times more CO, 11 times more formate, and 13 times more oxalate compared to equimolar amounts of the monomeric phenazine, demonstrating that the COF architecture enhances catalytic performance (TOF_COF_: 10^−7^ s^−1^ CO, 10^−8^ s^−1^ formate, and 10^−11^ s^−1^ oxalate). Structural analysis, including X‐ray diffraction and N₂ porosimetry, confirmed the COF's long‐range order and porosity. Mechanistic studies suggest a sequential formate‐to‐oxalate pathway, with CO and formate acting as intermediates. These results demonstrate the potential of the COF architecture to improve the performance of metal‐free, redox‐active aromatic systems such as phenazines to facilitate efficient and selective CO₂ conversion under mild conditions.

## Graphical abstract

The catalytic advantages of covalent organic frameworks (COFs) for sustainable CO₂ utilization have been demonstrated by the photocatalytic reduction of CO₂ by a redox‐active, phenazine‐based COF (Phen‐COF). Phen‐COF efficiently converts CO₂ into formate, oxalate, and carbon monoxide under light irradiation, achieving significantly higher yields than a comparable monomer.

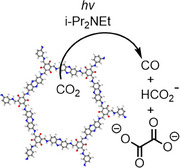

The electro‐ or photoreduction of CO_2_ transforms this greenhouse gas into valuable products, such as CO or hydrocarbons, providing fuels or precursors for fine chemicals.^[^ ^1^, ^2^, ^3^, ^4^, ^5^ ^]^ Historically, metallic catalysts and metal‐based materials such as copper,^[^ ^6^, ^7^, ^8^ ^]^ silver,^[^ ^9^ ^]^ and titanium dioxide^[^ ^10^, ^11^, ^12^ ^]^ have shown high effectiveness for CO_2_ reduction. Other promising systems include polyoxometalates^[^ ^13^, ^14^ ^]^ and metal‐organic frameworks.^[^ ^15^, ^16^, ^17^, ^18^, ^19^ ^]^ However, these catalysts rely on finite metal resources^[^ ^20^, ^21^ ^]^ and pose environmental risks from potential metal leaching.^[^ ^22^, ^23^ ^]^ In contrast, all‐organic systems, such as graphene oxide,^[^ ^24^, ^25^ ^]^ graphitic carbon nitride,^[^ ^26^ ^]^ and conjugated organic polymers^[^ ^27^, ^28^ ^]^ capitalize on abundant elements that, in principle, can be derived from renewable resources.^[^ ^29^, ^30^ ^]^ The structures of organic materials can be modified to establish structure‐property relationships to maximize quantum yield, turnover rate, and product selectivity,^[^ ^28^, ^30^, ^31^, ^32^, ^33^, ^34^, ^35^ ^]^ despite being less obviously suited for multielectron transfer processes associated with transition metals. Recently, integrating catalytically active groups within crystalline, permanently porous frameworks such as covalent organic frameworks (COFs)^[^ ^31^, ^32^, ^33^, ^36^, ^37^ ^]^ has become of interest because they adopt porous, ordered solid‐state structures whose structures are somewhat predictable based on the relative orientations of their polymerizable groups.

COFs are crystalline, porous polymers comprised of organic monomers that are covalently linked to form stacked, 2D sheets,^[^ ^38^, ^39^, ^40^, ^41^, ^42^ ^]^ or 3D networks providing periodic structures, high surface areas, and precise control over the spatial arrangement of reactive centers.^[^ ^31^, ^32^, ^33^, ^36^, ^37^, ^38^, ^41^, ^43^, ^44^ ^]^ Although some COFs containing metal complexes as building blocks have shown promising CO_2_ reduction capabilities,^[^ ^36^, ^45^, ^46^, ^47^ ^]^ a few metal‐free systems have demonstrated efficient performance under mild conditions.^[^ ^31^, ^32^, ^33^, ^48^, ^49^, ^50^ ^]^ Fu et al. utilized COFs comprised of azines, which facilitated efficient multielectron transfer to CO_2_ to generate CH_3_OH. Under irradiation at 420 nm, the azine COFs yielded a turnover frequency (TOF) of 0.0053 s^−^¹.^[^ ^33^ ^]^ Yu et al. reported high selectivity for CO generation in COFs that incorporated 4‐carboxyl‐quinoline linkages. Their high affinities for water and CO₂ resulted in a Faradaic efficiency of 97% for CO production and a TOF of 0.0080 s^−^¹ under irradiation using 420 nm light.^[^ ^50^ ^]^ Cui et al. employed bisthiazole‐containing, β‐ketoenamine‐linked COFs for CO₂ reduction. This system, under 400 nm light irradiation, achieved a Faradaic efficiency of 89% for CO generation with a TOF of 0.0043 s^−^¹.^[^ ^32^ ^]^ These notable examples of COF‐catalyzed CO_2_ reduction underscore how the selection of active sites can significantly influence catalytic activity and product outcomes.

Previously, we reported a phenazine‐based COF that exhibited desirable charge storage capacity because of its reversible redox processes.^[^ ^51^ ^]^ These properties make phenazines of interest for electrochemical processes, such as supercapacitors, redox flow batteries, and electro and photoredox catalytic systems.^[^ ^52^, ^53^, ^54^, ^55^ ^]^ Although molecular phenazines have been investigated for CO_2_ reduction, they typically require metal cocatalysts for significant activity.^[^ ^56^, ^57^ ^]^ Here, we report a phenazine‐based 2D COF that facilitates CO_2_ photoreduction without metal cocatalysts. This COF shows enhanced activity and selectivity for formate and oxalate, compared to a benzophenone‐containing phenazine monomer **1** (Figure 1). Under 390 nm irradiation, **Phen‐COF** achieved 2.9 times higher CO production, 11 times higher formate production, and 13 times higher oxalate production. We attribute this increased activity and selectivity to the COF architecture and its high local concentration of phenazine groups. These findings underscore the benefits of integrating photoactive units such as phenazines into COFs, thereby providing increased activity for metal‐free CO_2_ conversion.

**Figure 1:**
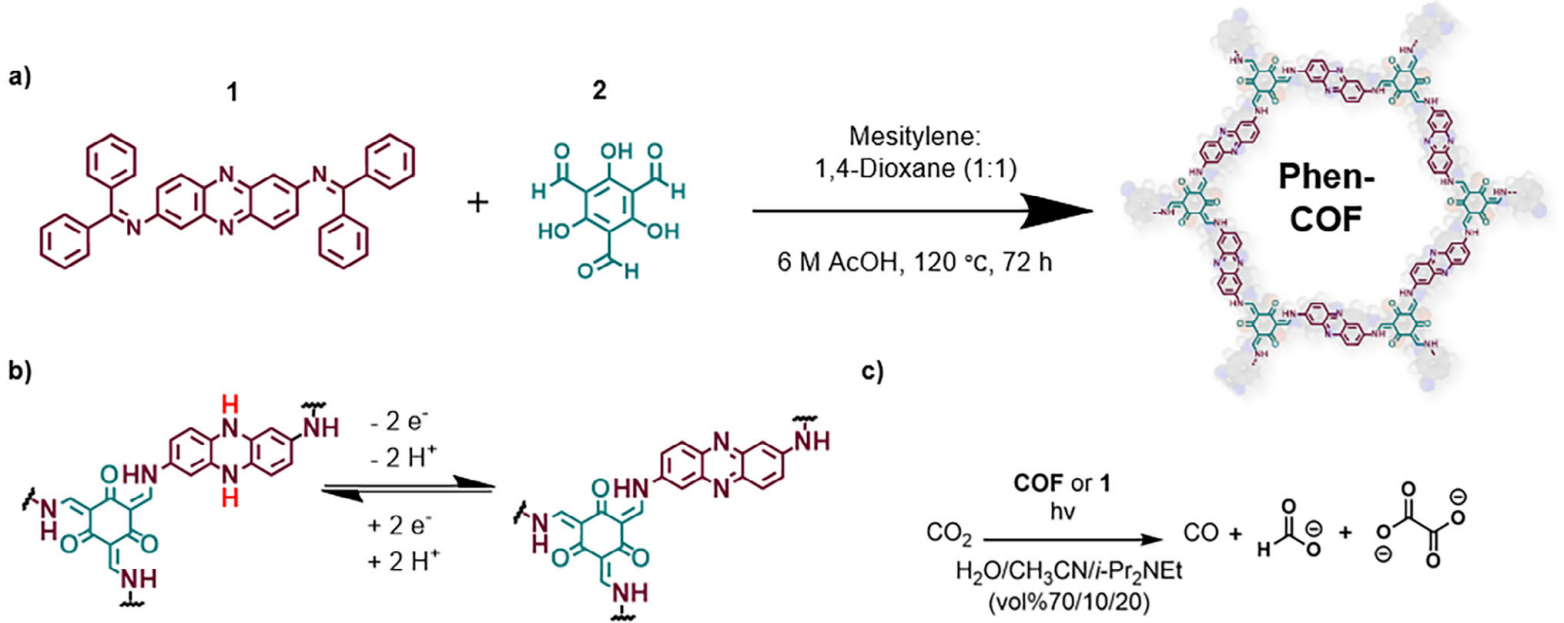
a) Solvothermal synthesis of **Phen‐COF** from the condensation of **1** and **2**. b) Schematic of the redox activity of **Phen‐COF** and 1 involved in c) the reduction of carbon dioxide to carbon monoxide, formate, and oxalate.

**Phen‐COF** was prepared by condensing **1** and **2** in the presence of CH_3_CO_2_H in a mixture of mesitylene:1,4‐dioxane (1:1 *v*/*v*) at 120 °C for 96 h. (see Supporting Information for detailed procedures).^[^ ^51^ ^]^ The crude COF powder was isolated by filtration and resuspended in DMF with stirring three times at 90 °C for 30 min to dissolve and separate residual phenazine monomer and oligomeric by‐products. After this washing procedure, the isolated COF powder was washed with ethanol and then acetone to remove most of the residual DMF from the pores. We assessed activation by supercritical drying, high vacuum at room temperature, and high vacuum at 120 °C (Figure S19). Drying at 120 °C under high vacuum proved to be the most effective for obtaining the **Phen‐COF** as a crystalline and porous powder, which we attribute to DMF being retained in the pores despite extensive washing with other solvents.

X‐ray diffraction and N_2_ porosimetry measurements indicated the long‐range order and permanent porosity of **Phen‐COF**. Powder X‐ray diffraction (PXRD) measurements showed a sharp ⟨100⟩ Bragg diffraction peak at 3.2°, consistent with a typical AA stacked average structure, slightly higher than the simulated Bragg peak due to residual solvent remaining in the pores (Figure 2a and Supporting Information Section VI). However, it is likely that this and other AA‐stacked COFs have significant stacking disorder based on recent studies of single‐crystalline 2D COFs.^[^ ^58^ ^]^ Weaker diffraction peaks at 5.8°, 6.5°, and 26.9° were observed and correspond to the ⟨110⟩, ⟨210⟩, and ⟨001⟩ reflections, respectively (Figure S14). **Phen‐COF** was confirmed to have a high thermal stability up to 400 °C using thermogravimetric analysis (Figure S13). N_2_ adsorption isotherms (Figure 2b) exhibited a Type 2 isotherm, which corresponded to a Brunauer−Emmett−Teller (BET) surface area of 1160 m^2^/g. Nonlocal density functional theory (NL‐DFT) analysis of the isotherm indicated a pore size distribution containing two peaks, with the first in the range of 21–23 Å, which is close to the theoretical value of 25 Å obtained from geometry‐minimized, eclipsed COF unit cells (Figure 2d). The second peak indicates smaller pores in the range of 18–20 Å. Although two similar pore sizes have local maxima in the NL‐DFT‐derived pore size calculations, it is unclear whether these arise from structural defects in the COF.

**Figure 2:**
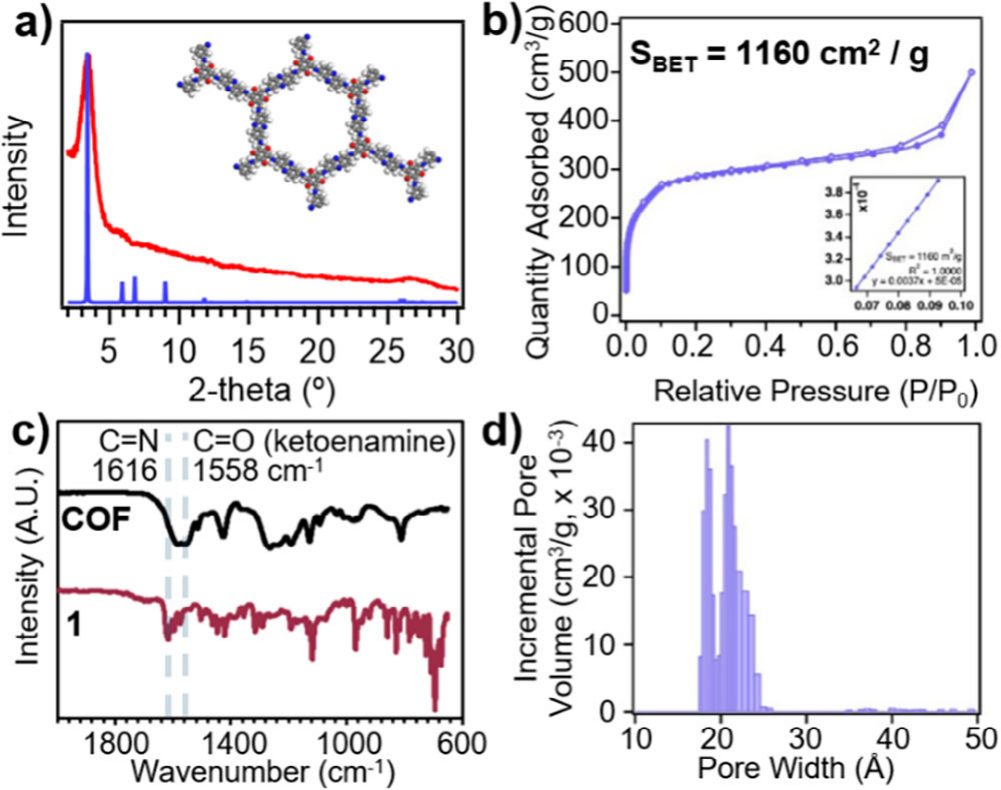
a) Powder X‐ray diffraction (red) of **Phen‐COF** powder. The simulated pattern of a theoretical AA‐stacked structure is also shown (blue). b) N_2_ isotherm of **Phen‐COF**. c) FTIR spectra of **Phen‐COF** and **1**. d) Pore size distribution of **Phen‐COF**.

FTIR spectroscopy (Figure 2c) of **Phen‐COF** confirmed the presence of β‐ketoenamine signals (C═O at 1600 cm^−1^ and C─N at 1250 cm^−1^).^[^ ^51^ ^]^ The COF signals are distinct from the spectrum of monomer **1** because of its benzophenone imine stretches (C═N at 1650 cm^−1^_,_ Figures 2c, S8). The ^13^C cross‐polarization magic‐angle spinning (CP‐MAS) solid‐state NMR spectrum of **Phen‐COF** showed resonances of C═O at ∼180 ppm, C═CNH at ∼145 ppm, and ═CNH at ∼115 ppm (Figure S4). In comparison, the solution ^13^C NMR spectrum of **1** shows similar resonances between 144 and 115 ppm (Figure S5), arising from the carbons in the phenazine heterocycle. Further, the specific atomic binding energies of the nitrogen, oxygen, and carbon atoms were determined by X‐ray photoelectron spectroscopy (XPS, Figures S10–S12). The N_1s_ XPS spectrum showed a peak shoulder at 398.5 eV, corresponding to the aromatic nitrogen on the phenazine, and 399.5 eV, corresponding to the C─N on the ketoenamine. Additionally, the C_1s_ XPS spectrum shows a small shoulder at 287 eV (C═O) and a larger shoulder at 285.5 eV (C─N), which is also consistent with a β‐ketoenamine linkage.^[^ ^59^ ^]^ Elemental analysis by ICP‐OES was consistent with the molecular formula of **Phen‐COF** and showed residual palladium content of the washed and activated COF sample was < 0.1 wt% (Supporting Information Section I).

Insight into electronic structure and optical properties reveals that both **Phen‐COF** and **1** have suitable optical absorption and energy levels for CO_2_ photoreduction. We analyzed the excited‐state reducing power of **Phen‐COF** by estimating the electronic excited state energy (*E*_op_) from the onset of absorption (Figure 3a). Tangent lines drawn to the lowest offset energy indicate an onset absorption energy of 2.50 eV for **1** and 2.58 eV for **Phen‐COF**. Assuming a direct energy transition, Tauc plot analysis yields similar values for the optical bandgap (*E*_g_) of 2.58 eV for **1** and 2.51 eV for **Phen‐COF** (Figure 3b). Although the COF structure imposes long‐range order, the electronic transitions remain largely confined to the phenazine units. Thus, this localized behavior limits the spectral redshift typically observed in extended conjugated systems. Binding energies determined by UV‐photoelectron spectroscopy (UPS, Figure 3c) show values below the Fermi level within the material bandgap, with tangent lines drawn to the lowest offset energy showing valence band maxima of 1.0 eV for **Phen‐COF** and 1.3 eV for **1** (Figure 3d). Together, these findings suggest that **Phen‐COF** and **1** can drive processes with standard reduction potentials ranging from −1.3 to 1.0 eV (Figure S27). Additionally, these valence band maxima confirm their ability to oxidize the sacrificial electron donor *i*‐Pr_2_EtN (0.8 eV in H_2_O/CH_3_CN).^[^ ^60^ ^]^ Since under basic aqueous conditions the standard reduction potentials for the conversion of CO_2_ to carbon monoxide is approximately −0.52 V versus SHE,^[^ ^1^, ^61^ ^]^ these results demonstrate that both **Phen‐COF** and **1** are thermodynamically capable of driving the processes for CO_2_ reduction.

**Figure 3:**
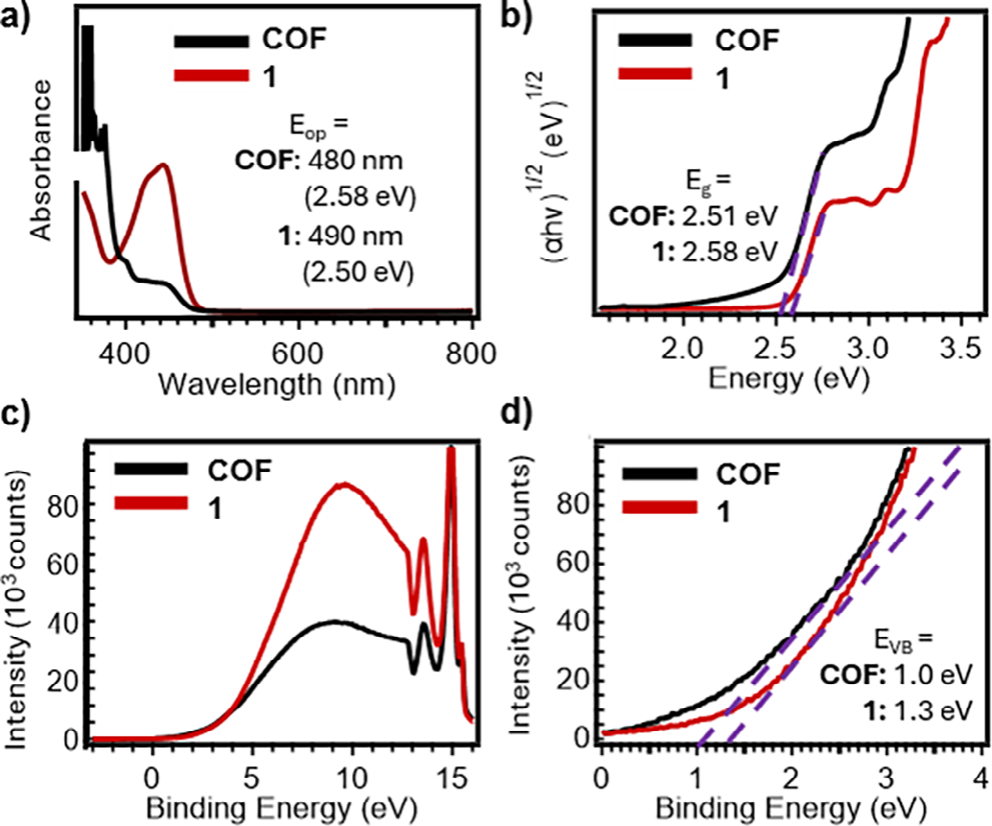
a) UV–vis spectrum of **Phen‐COF** and **1** (0.1 mM, acetonitrile containing 50 µM i‐Pr_2_NEt). b) Tauc plot of **Phen‐COF** and **1** with the extrapolated optical bandgap. c) Ultraviolet photoelectron spectrum for **Phen‐COF** and **1** with zoom‐in of the d) valence band maxima determined for **Phen‐COF** and **1**.

To evaluate and compare the photocatalytic reduction of CO_2_ in **Phen‐COF** and **1**, we designed and constructed a glass photoreactor equipped for gas‐line connections, enabling continuous flow gaseous monitoring through mass flow controllers (MFC, Figure 4a). The photoreactor is a transparent, 1 cm‐thick rectangular reaction chamber with 10 × 10 cm sides for efficient light penetration (Figure S24). Two fritted, lateral tubes protrude from the bottom and are designed with fritted Swagelok fittings to a gas flow cell. After purging with CO_2_, leaks in the system were minimized by leakage assessment methods until internal pressure losses were negligible. It was essential to ensure a properly sealed system, with all potential leaks addressed through rigorous leak detection and pressure monitoring. Experiments assessing materials for photocatalysis began with CO_2_ purging and GCMS monitoring until only CO_2_ was detected in the stream (detailed GCMS procedures and standardization in Supporting Information Section IX). The photoreactor was then exposed to UV irradiation (390 nm, Figure 4b, procedures in Supporting Information Section X), resulting in a greenish‐blue luminescence for **Phen‐COF** and a weak luminescence for **1**. In all CO_2_ reduction experiments, the amounts of each catalyst were chosen such that equimolar amounts of phenazines were used for **Phen‐COF** and **1**. The gas stream was recirculated through the photoreactor at a pressure of 20 psi with constant flow monitoring. A small sample of the circulating gas stream was injected into the GCMS every 15 min, which monitored CO_2_, CO, N_2_, and O_2_.

**Figure 4:**
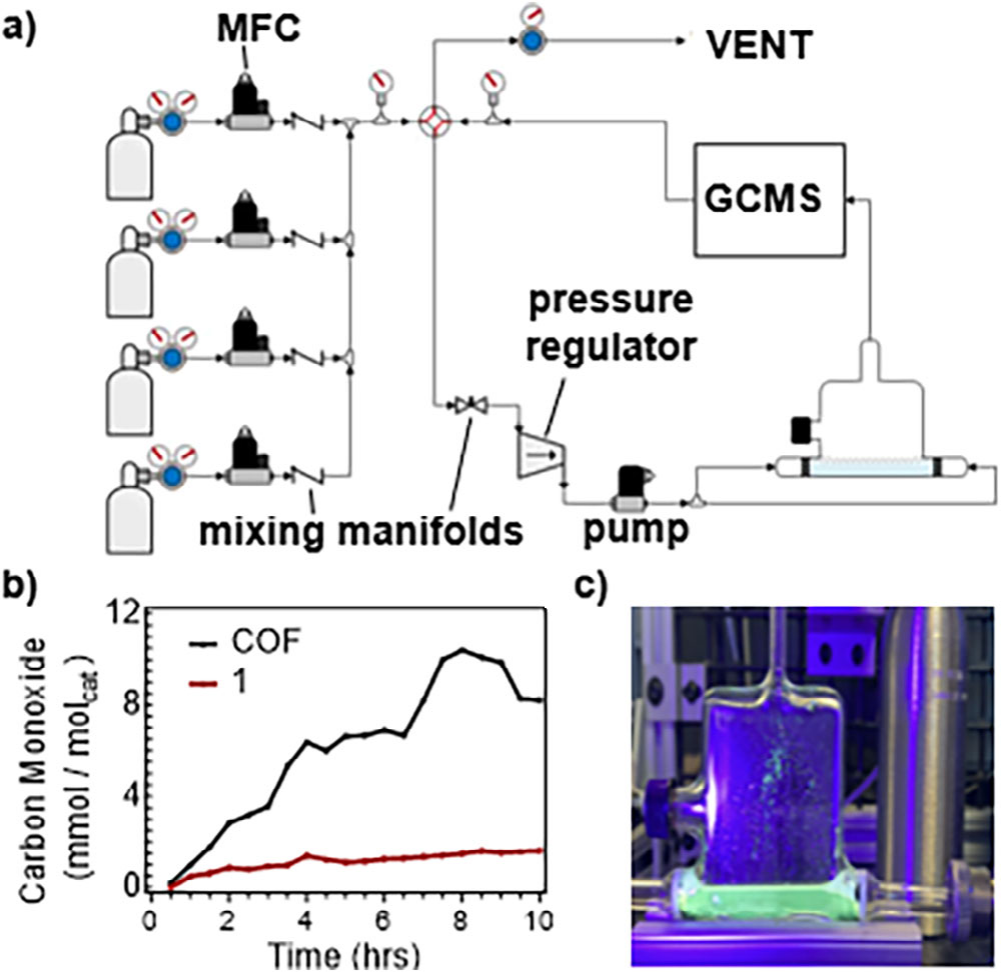
a) Schematic for gas line controllers and recirculating loop for *in operando* GCMS analysis. The flow rate into the custom glass photoreactor was maintained at 18.5 psi. b) Representative CO production traces over time for **Phen‐COF** and **1**. c) Optical image of the photoreactor containing **Phen‐COF** under irradiation at 390 nm.

Both **Phen‐COF** and **1** generated measurable CO within 30 min of irradiation (Figure 4b,c). CO generation was negligible in samples that were blocked from the UV source under the same conditions. Over 13 h of irradiation, **Phen‐COF** showed a TOF of 2.9 × 10^−7^ s^−1^ (0.95 mmol mol^−1^_cat_·h) outperforming the monomer's TOF of 1.0 × 10^−7^ s^−1^ (0.33 mmol mol^−1^_cat_·h). For both **Phen‐COF** and **1**, CO levels generally showed an upward trend between most measurements. Subsequent analysis of the catalyst slurry by ^13^C and ^1^H NMR spectroscopy indicated the formation of formate and oxalate (Figures S6,S7). We repeated the photocatalytic experiments using the same conditions, except for using visible light under irradiation at 467 nm (Figure S23). Similar to results at 390 nm, **Phen‐COF** displayed a TOF of 3.3 × 10^−7^ s^−1^, and **1** showed a TOF of 1.1 × 10^−7^ s^−1^.

Ion chromatography of aliquots of the reaction mixture characterized the formation of formate and oxalate during CO_2_ reduction. During photocatalysis by both **Phen‐COF** and **1**, aliquots of the liquid slurry were removed from the photoreactor through syringes with no losses in internal pressure and subjected to ion chromatography analysis (detailed standardization methods are provided in the Supporting Information). Peaks corresponding to formate (*t* = 3.9 mins, Figure 5a) and oxalate (*t* = 11 min, Figure 5b) indicated, after 13 h of photocatalysis under irradiation, **Phen‐COF** generated 11‐fold more formate and 13‐fold more oxalate compared to **1** after normalizing the data by moles of phenazine catalyst. Comparable amounts of oxalate and formate were also observed under irradiation at 467 nm (Figure S34).

**Figure 5:**
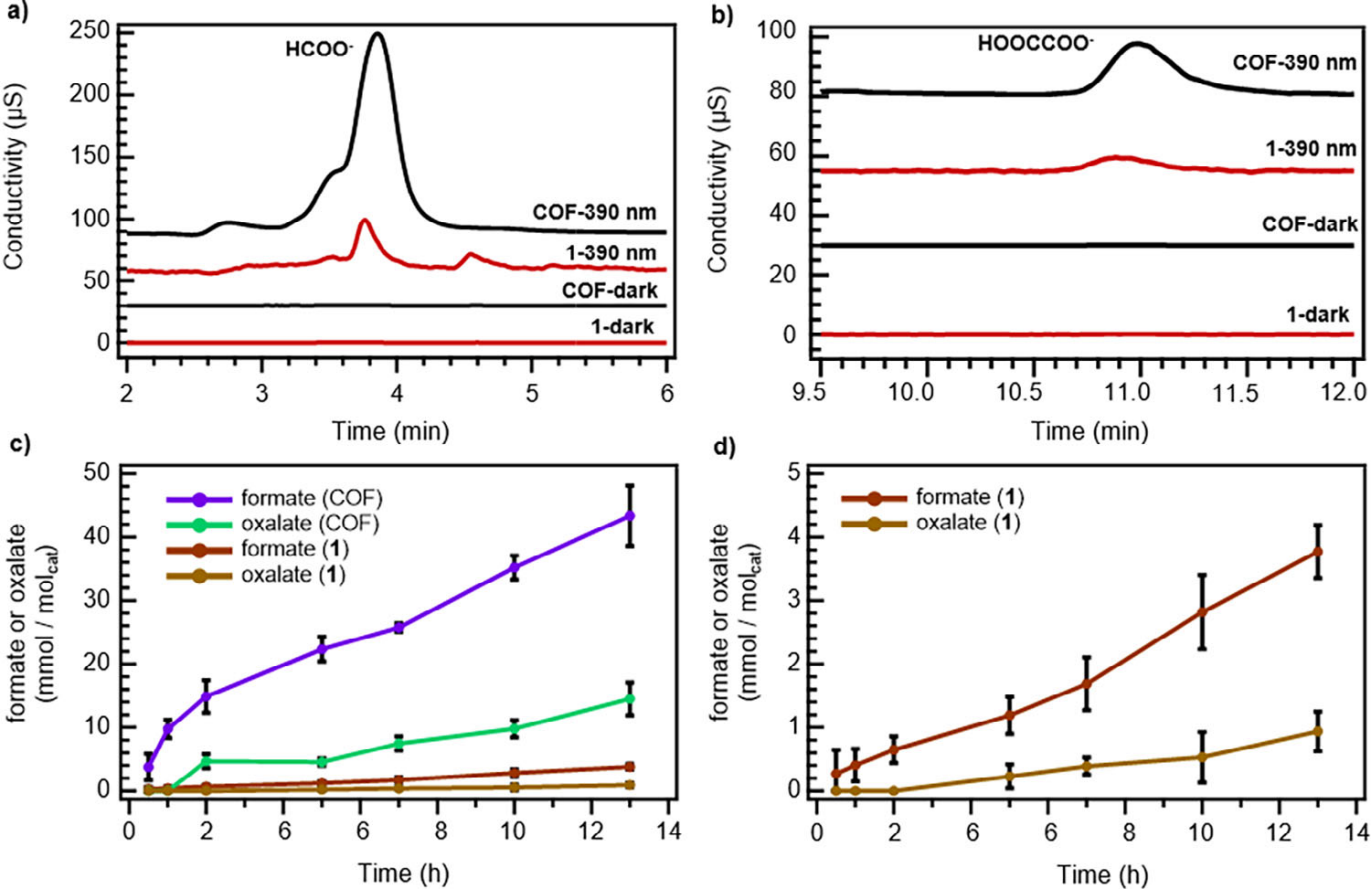
Ion chromatography traces of a) formate produced after 13 h by **Phen‐COF** and **1** and b) oxalate produced after 13 h by **Phen‐COF** and **1**, including traces of reactions from **Phen‐COF** and **1** after 13 h without irradiation. c) Determined amounts at varied time intervals by ex‐situ ion chromatography to quantify the amounts of formate and oxalate produced by **COF** and **1** throughout 13 h of irradiation (14.5 mM **Phen‐COF** or **1** in H_2_O/CH_3_CN/ i‐Pr_2_NEt {7/1/2, 2 mL}, *λ* = 390 nm, and Supporting Information Section X‐XI). d) Expansion of the plot in panel (c) to show the time‐dependent formation of formate and oxylate by **1**. Error bars in panels (c) and (d) depict the standard deviation of three measurements.

Time‐interval aliquot analysis of the liquid products during the first 13 h of photocatalysis revealed differences in product induction periods, suggesting the sequential generation of formate followed by oxalate. Ion chromatography traces for three photocatalytic trials over 13 h were quantified (Figure 5c,d). Turnover frequencies (TOFs) were calculated after 13 h with respect to the mols of phenazine in each experiment (Table 1). The results show that measurable amounts of formate are produced as early as 30 min in the reaction for both **Phen‐COF** and **1**. Although both **Phen‐COF** and **1** showed generation of formate and oxalate, **Phen‐COF** showed higher amounts of both species and began generating oxalate faster than **1**. Oxalate signals appeared after 2 h for **Phen‐COF** and 5 h for **1**. For **Phen‐COF**, the selectivity for CO, formate, and oxalate was 7.2%, 76.3%, and 16.5%, respectively. For monomer **1**, the selectivity for CO, formate, and oxalate was 55.3%, 39.9%, and 4.8%, respectively. These different product distributions (Figure S35) highlight the COF's higher selectivity for forming two‐carbon products. Since the limit of detection for oxalate was determined to be below 0.1 µmols, these results demonstrate an induction period necessary for oxalate generation that is not required for formate generation. Based on these observations, we hypothesized that oxalate is produced from formate under the reaction conditions, which we evaluated in follow‐up experiments.

**Table 1:** TOFs of products formed during photocatalytic CO_2_ reduction.

	CO (s^−1^)	Formate (s^−1^)	Oxalate (s^−1^)
**Phen‐COF**	2.9 × 10^−7^	7.1 × 10^−8^	2.0 × 10^−11^
**1**	1.0 × 10^−7^	6.2 × 10^−9^	1.5 × 10^−12^

Mechanistic studies of **Phen‐COF** reveal that both carbon monoxide and formate contribute to the formation of downstream products, following a sequential pathway from formate to oxalate. Photocatalytic experiments using carbon monoxide as the starting material, rather than carbon dioxide, were conducted in sealed, stirred flasks (Figure 6a). Liquid product analysis showed a rapid production of formate at early time points, analogous to CO_2_‐driven reactions, although the overall evolution rate was significantly lower with CO as the starting material (0.036 mmol mol^−1^_cat_·hr). Control reactions sealed under an atmosphere of nitrogen instead of CO_2_ demonstrated no generation of formate or oxalate, indicating that these products are not formed from degradation of the catalyst or sacrificial electron donor (iPr_2_EtN, *N*,*N*‐diisopropylethylamine). Oxalate production was again minimal until 5 h into the reaction, indicating that oxalate is not directly produced from CO, making it likely that the oxalate originates from the produced formate. This hypothesis was confirmed in subsequent photocatalytic experiments that used sodium formate as the starting material under a nitrogen atmosphere (Figure 6b). Under these conditions, oxalate was produced more rapidly and in greater amounts compared to when CO_2_ or CO were used as reactants. Taken together, several observations support a mechanism in which oxalate is produced from formate under the same conditions that CO_2_ and CO undergo photoreduction. Furthermore, although carbon monoxide produces formate and oxalate, this reaction occurs more slowly than CO_2_ reduction and is likely a small contributor to product formation when CO_2_ is present in significant amounts.

**Figure 6:**
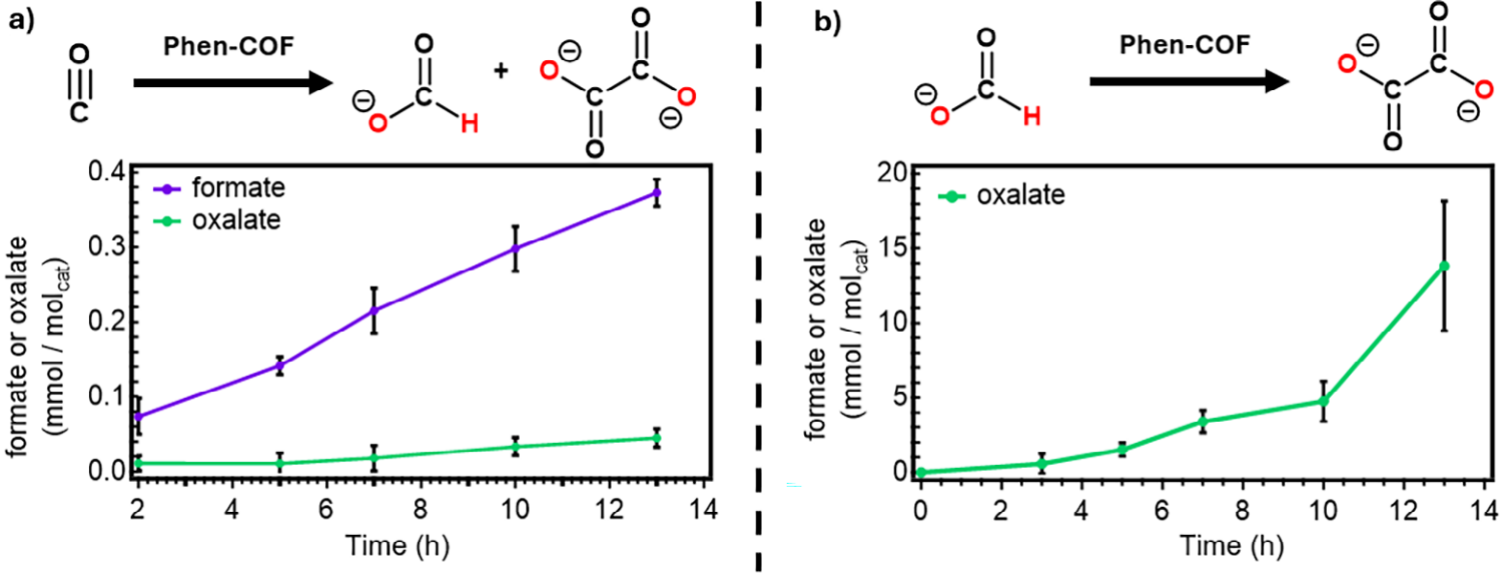
a) Formate and oxalate produced from the photocatalytic reduction of carbon monoxide using **Phen‐COF**. b) Oxalate produced from the photocatalytic reduction of formate under a nitrogen atmosphere using **Phen‐COF**. (40 mM of **Phen‐COF** H_2_O/CH_3_CN/i‐Pr_2_NEt. {7/1/2}, *λ* = 390 nm, 18.5 psi, and 10 m.) Error bars depict the standard deviation of three measurements.

CO_2_ reduction activity of **Phen‐COF** and **1** depended on the presence of i‐Pr_2_NEt, a solvent mixture of H_2_O/CH_3_CN/ i‐Pr_2_EtN (7/1/2), light irradiation, and CO_2_ (Figures S25,S26). These experiments are consistent in prior literature studies on phenazine‐assisted CO_2_ reduction, in which an excited phenazine is reductively quenched to a phenazine radical anion, followed by protonation to a phenizyl radical, which undergoes electron transfer to CO_2_ (Figure S36).^[^ ^38^ ^]^ Although phenazine‐catalyzed oxalate production could involve carbonite ion (CO_2_^2−^) intermediates from the reduction of formate, we cannot make any definitive claims.^[^ ^62^ ^]^ The porosity and high local phenazine concentration of **Phen‐COF** significantly boosts product generation and alters the product distribution to favor oxalate production. Although **Phen‐COF** only converts 1.87% of CO_2_ and appears less active than previously reported metal‐free COF catalysts (Table S2) and metal‐free photocatalysts (Table S3) for CO_2_ reduction, the selectivity for components like formate and oxalate is unique and potentially relevant for obtaining even higher carbon‐number compounds from CO_2_ in the future. However, potential scalability challenges and boosting performance under wider environmental conditions are still important limitations compared to emerging organic semiconductor materials. Postreaction PXRD analysis (Supporting Information Figure S20) indicates that **Phen‐COF** retains its crystallinity after prolonged photocatalytic testing, demonstrating good photostability under the reaction conditions. A reduction in crystallinity as judged by the broadening of the ⟨100⟩ peak, is observed after 16 h (post 1st cycle) and 32 h (post 2nd cycle). After 32 h of photoirradiation, isolation, and activation of the material under vacuum at 120 °C, the ⟨100⟩ peak sharpened, indicating retention of the material's crystallinity. The differences among these patterns may correspond to slight differences in layer stacking that occur upon suspension in various solvents or as a result of the 2nd activation procedure. FTIR spectroscopy after 32 h of exposure to catalytic conditions also indicates preservation of the β‐ketoenamine stretches (Figure S9).

In summary, we report the photocatalytic reduction of CO₂ to CO, formate, and oxalate using phenazines integrated within a β‐ketoenamine‐linked COF. We note significant differences in product distribution and activity for the COF photocatalyst as compared to its monomeric phenazine. Both **Phen‐COF** and **1** exhibited activity in generating formate and oxalate under photocatalytic conditions, but **Phen‐COF** demonstrated significantly higher turnover frequencies (TOF) and selectivity, particularly for oxalate. This enhanced performance is attributed to the increased surface area, improved charge transport, and spatial arrangement of phenazine units within the COF's eclipsed structure. Through UV–vis spectroscopy and UV–vis photoelectron spectroscopy, we determined the adsorption onset potentials and valence band maxima for both systems. Under 390 nm irradiation, **Phen‐COF** achieved a TOF of 2.9 × 10^−7^ s^−1^ (0.95 mmol mol^−1^_cat_·hr) outperforming the monomer's TOF of 1.0 × 10^−7^ s^−1^ (0.33 mmol mol^−1^_cat_·hr). Additionally, **Phen‐COF** generated formate and oxalate ten times faster than the monomer, underscoring its greater efficiency in downstream product formation. For the COF, the reductive dimerization of formate appears to be the most likely oxalate formation mechanism.

These findings underscore the critical influence of structural factors, such as porosity and electronic configuration, in enhancing the efficiency of catalytic CO₂ reduction. The increased accessibility of redox‐active sites in **Phen‐COF** provides a clear advantage over monomeric analogues. Furthermore, the significant boost in formate and oxalate production in **Phen‐COF** not only highlights its efficiency but also points to its potential in facilitating complex multielectron transfer sequences in COFs. By incorporating redox‐active phenazine uniformly throughout the COF, particularly in controlling the spatial arrangement of active sites, this work contributes to the growing interest in metal‐free catalytic materials for sustainable CO₂ reduction processes.

The authors have cited additional references within the Supporting Information.^[^ ^33^, ^59^, ^60^, ^62^, ^63^, ^64^, ^65^, ^66^, ^67^, ^68^, ^69^, ^70^, ^71^ ^]^

## Conflict of Interests

The authors declare no conflict of interest.

## Supporting information



Supporting Information

## Data Availability

The data that support the findings of this study are available in the supplementary material of this article.
