# Supplementary material for: A Phenazine‐Based Two‐Dimensional Covalent Organic Framework for Photochemical CO2 Reduction with Increased Selectivity for Two‐Carbon Products
Source: Angew Chem Int Ed Engl. 2025 Mar 18;64(21):e202502799. doi: 10.1002/anie.202502799 (PMC12087818; doi:10.1002/anie.202502799)
Supplement: Supplementary file 1 — Supporting Information [file ANIE-64-e202502799-s001.pdf]

Supplementary Information for:

A Phenazine-Based Two-Dimensional Covalent Organic Framework for Photochemical CO_2 Reduction with Increased Selectivity for Two-Carbon Products

Zoheb Hirani,[a] Neil M. Schweitzer,[b] Edon Vitaku,[c] and William R. Dichtel*[a]

[a] Department of Chemistry
[b] Department of Chemical and Biological Engineering,
Northwestern University 2145 Sheridan Road, Evanston, IL 60208 USA

Correspondence Address
Prof. William R. Dichtel<br>Department of Chemistry<br>Northwestern University<br>2145 Sheridan Road<br>Evanston, IL 60208 (USA)<br>wdichtel@northwestern.edu

WILEY - VCH

Table of Contents

Materials and Instrumentation 3
I. Synthetic Procedures 5
II. NMR Spectra 9
III. FT-IR Spectra 14
IV. X-Ray Photoelectron Spectroscopy 16
V. Thermogravimetric Analysis 18
VI. Simulation of X-ray Diffraction Patterns 19
VII. Powder X-ray Diffraction Data 24
VIII. Surface Area Measurements with BET insets 26
IX. GCMS Methods and Data 27
X. Photocatalytic Measurements 29
XI. Ion Chromatography Methods and Data 33
XII. Comparison to Other COF Photocatalysts 39

WILEY-VCH

Materials and Instrumentation

Materials

All reactions were performed using flame-dried glassware under an atmosphere of nitrogen with dry solvents, unless otherwise stated. Dry tetrahydrofuran (THF), acetonitrile (MeCN/CH_3CN), and toluene were obtained by passing previously degassed solvents through activated alumina columns over an Argon-filled solvent system. All carbon dioxide and carbon monoxide were research-grade (>99.999%). All other reagents were purchased from commercial sources and used without further purification, unless otherwise stated.

Instrumentation

Nuclear Magnetic Resonance Spectroscopy. ^{1}H and ^{13}C NMR spectra were acquired on Bruker Avance III-600 MHz equipped with a 5mm DHC with Z-Gradient CryoProbe and recorded at 25 °C with a 0 Hz spin rate. The spectra were calibrated using residual solvent as internal reference ($CDCl_3$: 7.26 ppm for ^{1}H NMR, 77.00 for ^{13}C NMR). The following abbreviations (or combination thereof) were used to explain multiplicities: s = singlet, d = doublet, q = quartet, p = pentet, m = multiplet, b = broad. Solid-state ^{13}C CP/MAS NMR spectra were recorded on a Bruker AvanceIII- 400 MHz equipped with a 4mm HX probe at a spin rate of 15,000 Hz and calibrated using Adamantane as an external standard.

Fourier Transform Infrared Spectroscopy. Infrared spectra were recorded on a Nicolet iS10 FT-IR Spectrometer equipped with a ZnSe ATR attachment and are uncorrected.

High Resolution Mass Spectrometry. High-resolution mass spectra were acquired on Agilent 6210A LC-TOF Mass Spectrometer, with Atmospheric Pressure Photoionization (APPI) as an ionization source. The instrument is equipped with an Agilent Series 1200 HPLC binary pump, and Autosampler, using Mass Hunter software. The samples were run using direct injection.

Powder X-ray Diffraction. Powder X-ray diffraction (PXRD) patterns were obtained at room temperature on a STOE-STADI P powder diffractometer equipped with an asymmetric curved Germanium monochromator (Cu$K_{\alpha 1}$ radiation, λ = 1.54056 Å) and one-dimensional silicon strip detector (MYTHEN2 1K from DECTRIS). The line focused Cu X-ray tube was operated at 40 kV and 40 mA. The as-obtained powder samples were sandwiched between two acetate foils mounted in flat plates with a disc opening diameter of 8 mm and measured in transmission geometry in a rotating holder. The instrument was calibrated against a NIST Silicon standard (640d) prior to the measurement.

WILEY-VCH

Gas Chromatography/Mass Spectrometry. GCMS measurmeents were conducted on an Agilent 7890A GC system utilizing an FID. Quantitation of CO, CO_2, and O_2 was carried out using ion chromatographic integrations at m/z = 12 (CO) and m/z = 44 (CO_2).

X-Ray and Ultraviolet Photoelectron Spectroscopy. X-ray Photoelectron Spectroscopy (XPS) data were collected using a Thermo Scientific NEXSA G2, equipped with ultraviolet photoelectron spectroscopy (UPS).

Thermogravimetric Analysis. Thermogravimetric analyses were performed using a Netzsch Simultaneous Thermal Analysis (STA, 449 F3 Juniper) instrument. Samples were weighed and loaded onto a 0.35 mL Alumina crucible. All crucibles were weighed separately and samples were measured under UHP-grade Helium gas (flow of 50 mL/min). Empty curcible measurements under the same conditions were taken for buoyancy effect corrections. Temperature was increased at a rate of 10 °C/min.

Elemental Analysis. Elemental analyses were performed by Robertson Microlit Laboratories using inductively-coupled plasma mass spectrometry (ICP-MS) for the quantification of carbon, hydrogen, nitrogen, and palladium content in the materials.

Gas Adsorption. Gas adsorption isotherms were conducted on a Micromeritics ASAP 2420 Accelerated Surface Area and Porosity Analyzer. Typically, 20-50 mg samples were transferred to dried and tared analysis tubes equipped with filler rods and capped with a Transeal. The samples were heated to 40 °C at a rate of 1 °C/min and evacuated at 40 °C for 20 min, then heated to 100 °C at a rate of 1 °C/min heat, and evacuated at 100 °C until the outgas rate was ≤0.3 μmHg/min (holding the samples at 100 °C for 5–8 h was sufficient), at which point the tube was weighed again to determine the mass of the activated sample. The tube was then transferred to the analysis port of the instrument. UHP-grade (99.999% purity) N_2 was used for all adsorption measurements. N_2 isotherms were generated by incremental exposure to nitrogen up to 760 mmHg (1 atm) in a liquid nitrogen (77 K) bath. Oil-free vacuum pumps and oil-free pressure regulators were used for all measurements. Brunauer-Emmett-Teller (BET) surface areas were calculated from the linear region of the N_2 isotherm at 77 K within the pressure range P/P_0 of 0.05 – 0.10. All BET linear fits had a minimum R^2 value of 0.999.

WILEY-VCH

I. Synthetic Procedures

Synthesis of Monomers.

Triformylphloroglucinol (**2**) was synthesized following a previously reported procedure, and the obtained NMR data is consistent with those reported in the literature.[1] 2,7-diaminophenazine·benzophenoneimine **(1)** was prepared via a three-step protocol (Scheme S1 and Scheme S2) using commercially available starting materials. **2,7-dibromophenazine** was prepared from a two-step procedure adapted from a previously published method.[2] Importantly, a key precursor was obtained and purified in Vitaku et al. (**S1,** 4-bromo-N-(4-bromophenyl)aniline), which was previously thought to be 4-bromo-2-nitro-N-(4-bromophenyl)aniline.[2] The final synthetic step to acquire **1** involves a one-pot Buckwald coupling followed by recrystallization (Scheme S2).

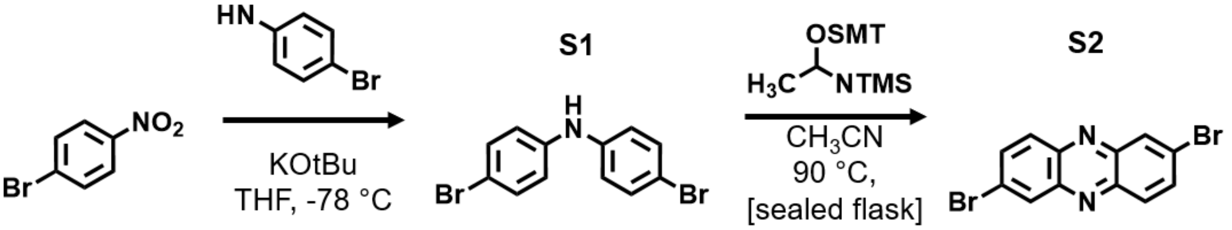

Scheme S1: Synthesis of phenazine precursor **S2** from 4-bromoaniline and 1,4-bromo-nitrobenzene.

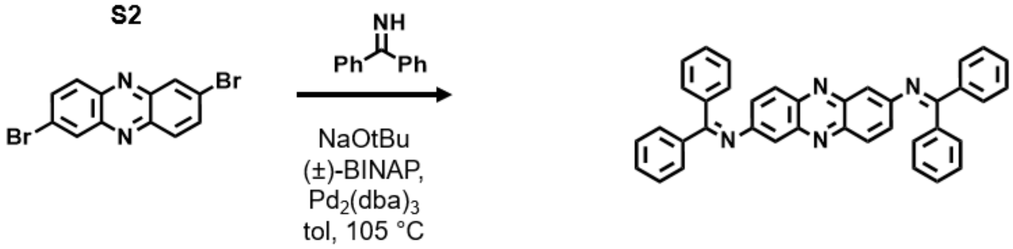

Scheme S2: Synthesis of **1** from **S2**.

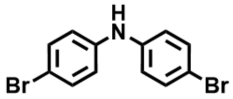

4-bromo-N-(4-bromophenyl)aniline (S1): To a 500 mL round-bottom flask, potassium t-butoxide (4.90 g, 43.61 mmol, 3.00 equiv) and THF (55 mL) were added, the flask was cooled to –78 °C (dry ice / acetone), and stirred for c.a. 10 min. 4-bromoaniline (2.50 g, 14.53 mmol, 1.00 equiv) dissolved in THF (15 mL) was added dropwise via a syringe (c.a. 5 min), followed by 1-bromo-4-nitrobenzene (2.93 g, 14.53 mmol, 1.00 equiv) dissolved in THF (30 mL) also added dropwise via a syringe (c.a. 5 min). The reaction was stirred at –78 °C for 2 hrs, and the reaction mixture was poured into a stirring solution of saturated ammonium chloride (175 mL). The product was extracted with EtOAc (3 x 75mL), and the organics were combined, washed with water (150 mL) and brine (150 mL). The organics were collected, dried with anhydrous magnesium sulfate, filtered, purified by column chromatography (direct solid-loading). The residue was purified via flash column chromatography using a 5-10% EtOAc/hexanes gradient over 15 mins. All fractions containing **S1** (red band; R_f = 0.15 in 10% EtOAc/hexanes) were collected to give the product (1.81 g, ~95% purity, 24% yield) as a red powder.

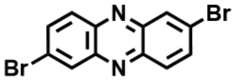

2,7-dibromophenazine (S2): To a 60-100 mL high-pressure or sealed schlenk flask, **S1** (1.81 g, 5.50 mmol, 1.00 equiv – assume pure for equiv calculations), N,O-bis(trimethylsilyl)acetamide (6.2 mL, 25.5 mmol, 5.0 equiv), and MeCN (20 mL) were added. The flask was sealed, and the reaction was stirred at 90 °C for 16 hrs. The flask was then gradually cooled to RT and the precipitate was collected via filtration and further washed with EtOH (~20 mL) to give crude **S2** (yellow solid, 1.32 g, 72% yield, >98%), which was used in the next step without any further purification.

^{1}H NMR (500 MHz, CDCl$_3$) δ 8.4 (d, J = 2.1 Hz, 2H), 8.1 (d, J = 9.2 Hz, 2H), 7.9 (dd, J = 9.2, 2.1 Hz, 2H).

^{13}C NMR (126 MHz, CDCl$_3$) δ 143.6, 142.3, 134.9, 131.7, 130.9, 125.4.

Characterization data is consistent with those previously reported.[2]

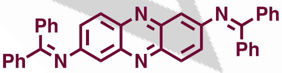

2,7-diaminophenazine·benzophenoneimine (1): To a 250 mL round-bottom flask, (±)-BINAP (0.70 g, 1.12 mmol, 0.30 equiv), tris(dibenzylideneacetone)dipalladium(0) (0.35 g, 0.38 mmol, 0.10 equiv), and toluene (70 mL, degassed) were added. The reaction mixture was purged with nitrogen over 30 min at RT. The reaction mixture was then stirred under reflux for 1 hr, cooled to RT, and under a nitrogen atmosphere, solid sodium t-butoxide was added(0.900 g, 10.00 mmol, 2.80 equiv), followed by solid **S2** (1.32 g, 3.75 mmol, 1.00 equiv), and benzophenone imine (1.63 g, 9.00 mmol, 2.40 equiv) dissolved in toluene (3 mL). More toluene (10 mL) was added to wash the walls of the

flask, and the reaction mixture was again purged for 30 minutes, heated and stirred under reflux for 16 hrs. The reaction mixture was cooled to RT and toluene was removed in vacuo. To this residue, EtOH (~300 mL) was added, the slurry was stirred at ~80 °C for 30 min, and the solid was collected via hot filtration to give crude **1** (yellow solid, NMR purity of crude >98%). The crude product was then purified via recrystallization (~4.5 g crude in a mixture of ~1:1 EtOH : $CHCl_3$, ~850 mL): Once dissolved from hot solvent, the solution was allowed to slowly cool to RT and then stored in a freezer to afford the product as a bright yellow solid (3.49 g, 86% yield).

^{1}H NMR (500 MHz, $CDCl_3$) δ 7.91 (d, J = 9.1 Hz, 2H), 7.82 (m, 4H), 7.53 – 7.44z (m, 6H), 7.35 (d, J = 2.2 Hz, 2H), 7.28 (dd, J = 9.1, 2.3 Hz, 2H), 7.23-7.18 (m, 10H).

^{13}C NMR (126 MHz, $CDCl_3$) δ 169.36, 152.47, 143.12, 141.31, 138.96, 135.62, 131.25, 129.60, 129.30, 129.03, 128.31, 128.21, 127.97, 116.30.

MS (ESI)$^+$ *m/z* calculated for $C_{38}H_{27}N_4$ $[M+H]^+$: 539.223; found: 538.985.

Elemental Analysis calculated for $C_{38}H_{26}N_4$: C 84.73%, H 4.87%, N 10.40%; found: C 84.51%, H 4.90%, N 10.23% Pd 0.0083%.

WILEY-VCH

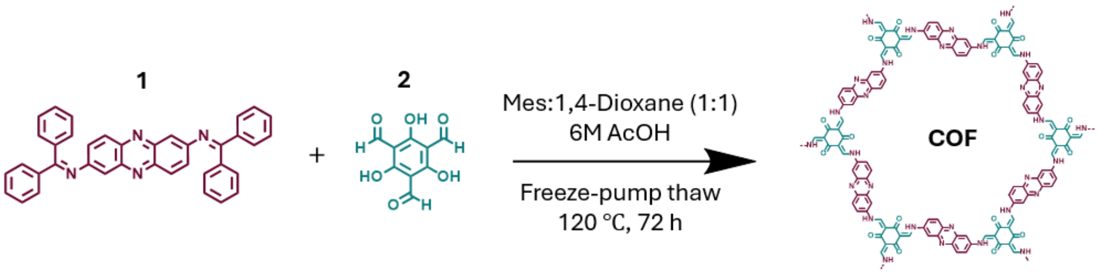

Scheme S3. Solvothermal synthesis of **Phen-COF**.

Phen-COF: In a 250 mL high-pressure flask equipped with a vacuum valve, monomer **1** (1.61 g, 3.0 mmol, 1.5 equiv) and monomer **2** (0.42 g, 2.0 mmol, 1.0 equiv) were added. A mixture of mesitylene:1,4-dioxane (1:1, 20 mL) was poured along the walls of the flask to help wash down residual solids. The flask was sealed and sonicated at room temperature for 10 min. Then, 6M acetic acid (3.3 mL) was directly added to the reaction mixture, followed by degassing through three freeze-pump-thaw cycles until a vacuum <50 mTorr was achieved. The flask was charged with N_2 and sealed under positive N_2 pressure (~1–2 psi). To avoid any unwanted benzophenone-induced side reactions, the flask was covered with aluminum foil and placed in an oil bath at 120 °C without stirring for four days.

After the reaction, the flask was removed from the oil bath, cooled to room temperature, and the mixture was filtered through a Buchner funnel with filter paper. Acetone and DMF were used to ensure complete transfer of material from the flask to the funnel. of the material is filtered from the flask to the Buchner funnel. The solid was then vigorously stirred in hot DMF (150 mL at 90 °C for 30 min) and filtered while still hot. This process was repeated two more times in DMF (150 mL at 90 °C for 30 min). Subsequently, the solid was washed in absolute ethanol (150 mL at 80 °C for 30min), and finally in acetone (300 mL at 60 °C). The material was then filtered, collected in a tared vial, and dried at 120 °C under vacuum (~50 mTorr) over 24 hrs to give DAPH-TFP COF as a reddish purple solid (1.72 g, 91% yield).

Solid-state CP/MAS ^{13}C NMR (101 MHz). δ 183.89, 140.09,128.39, 116.28, 107.47, 100.36.

Elemental Analysis calculated for $C_{27}H_{15}N_6O_3$: C 68.79%, H 3.21%, N 17.83%; found: C 68.18%, H 3.44%, N 17.03%, Pd 0.0096%.

WILEY-VCH

II. NMR Spectra

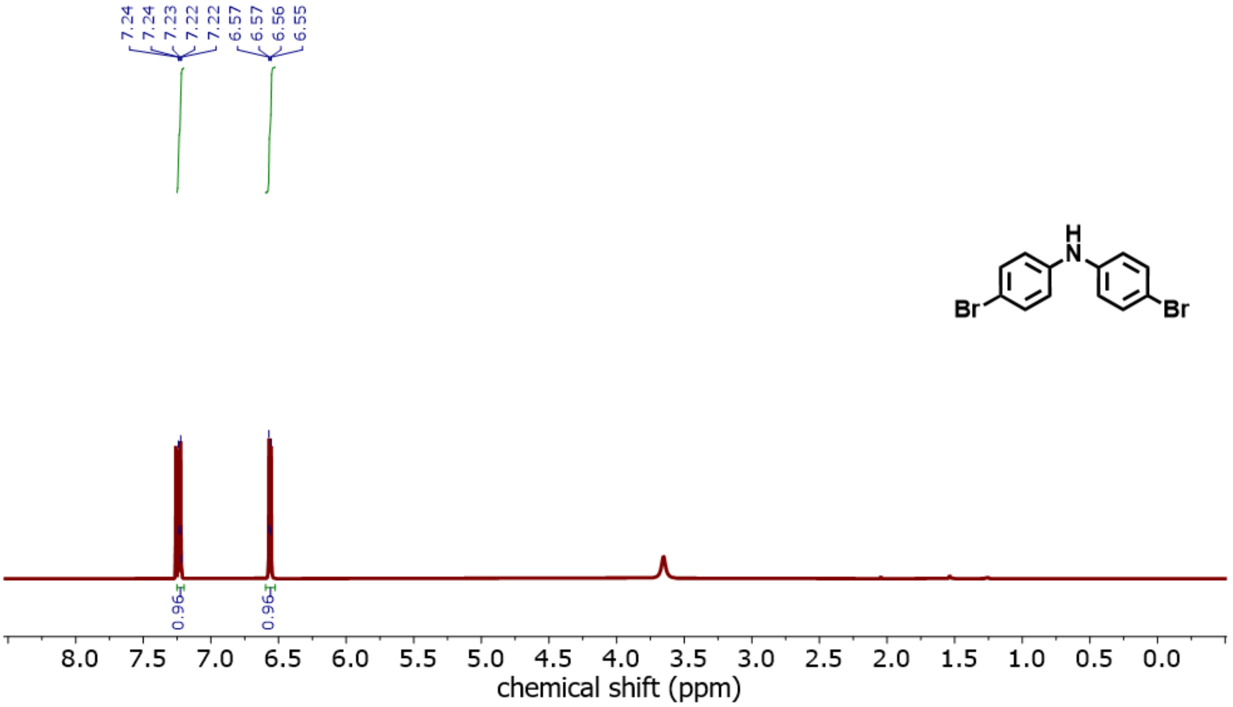

Figure S1: ^{1}H NMR of 4-bromo-N-(4-bromophenyl)aniline (**S1)** ($CDCl_3$, 500 MHz).

WILEY

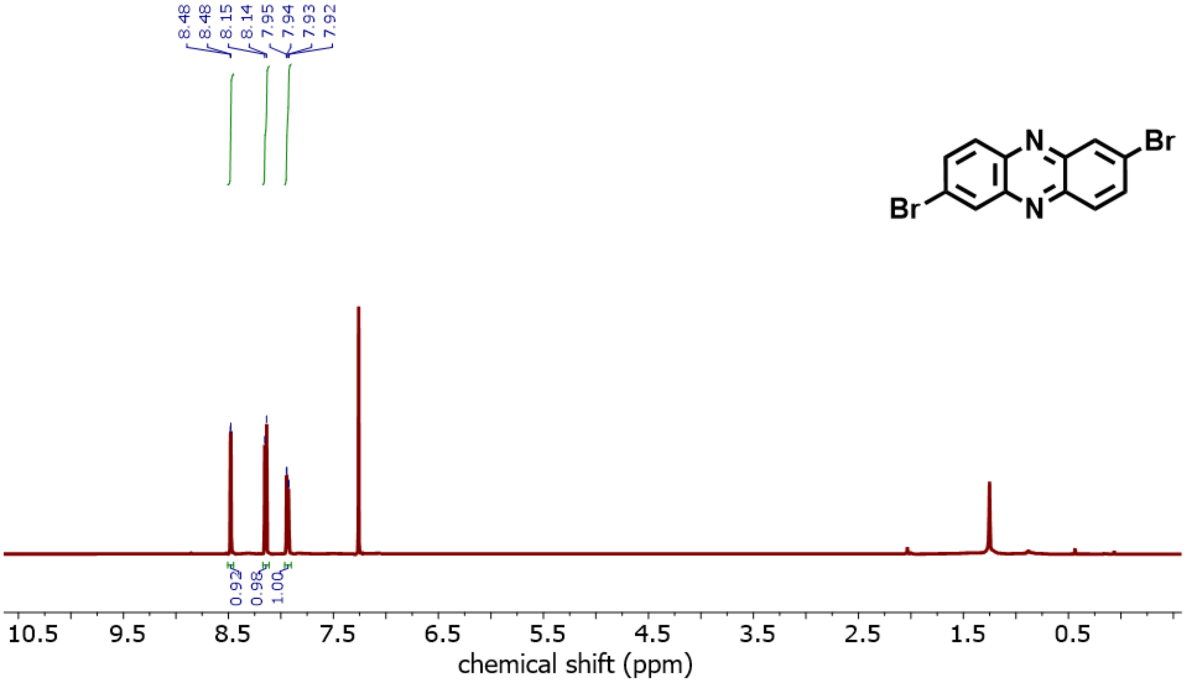

Figure S2. ^{1}H NMR of 2,7-dibromophenazine (**S2**) ($CDCl_3$, 500 MHz).

.

WILEY

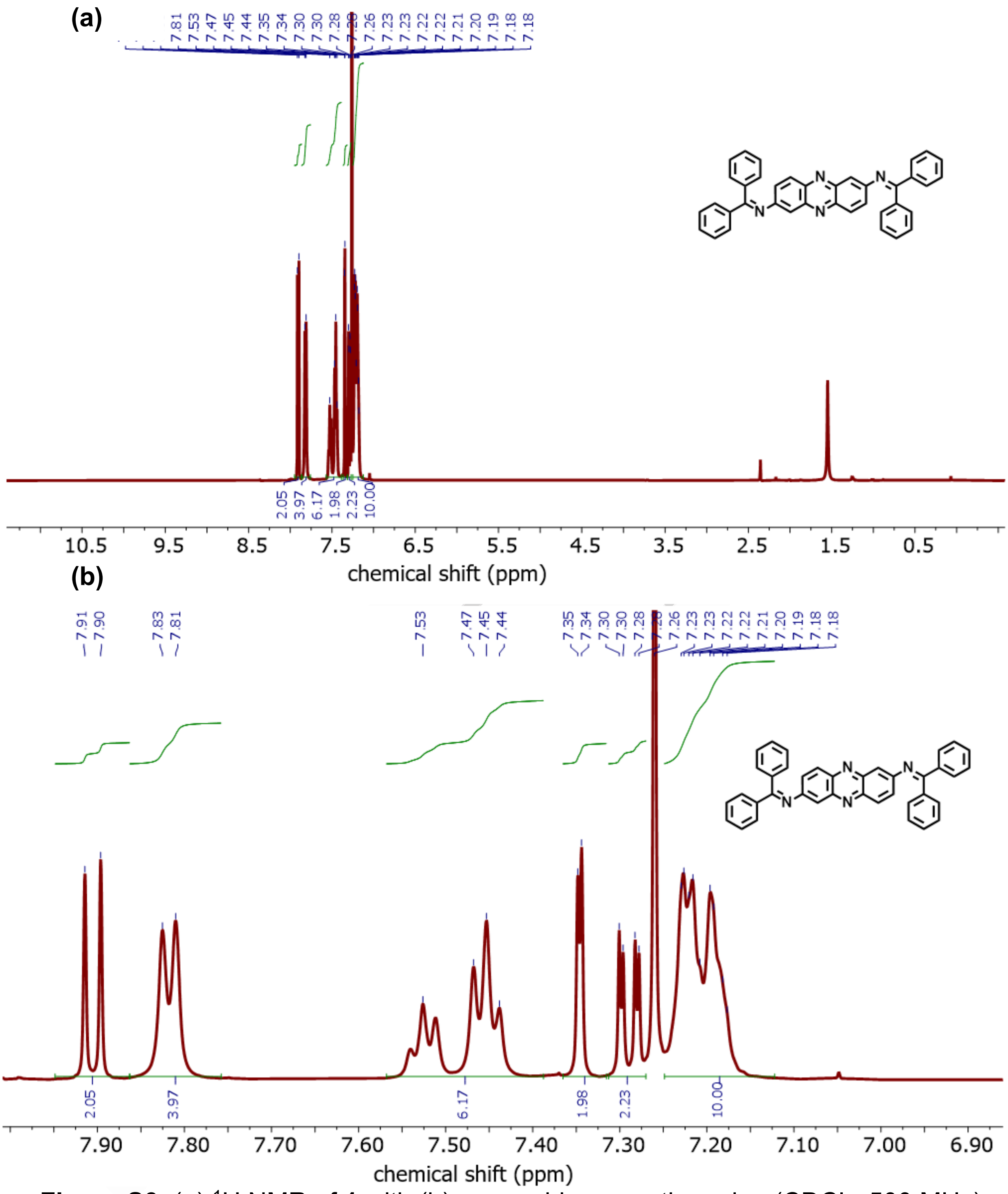

Figure S3. (a) ^{1}H NMR of **1** with (b) zoomed-in aromatic region ($CDCl_3$, 500 MHz).

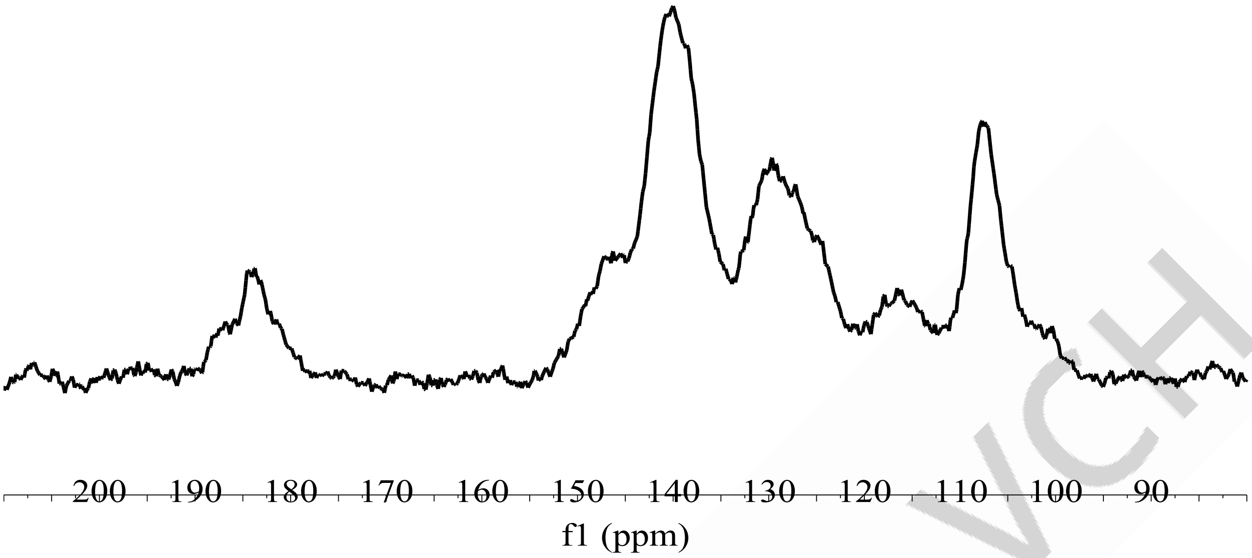

Figure S4. Cross-polarization magic-angle spinning (CPMAS) ^{13}C NMR of **Phen-COF.**

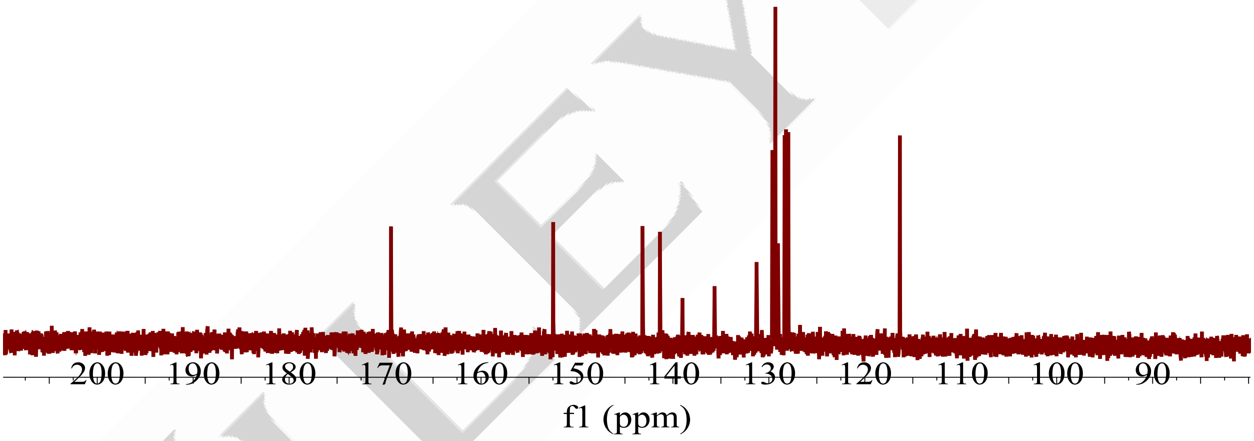

Figure S5. Solution-state ^{13}C NMR of **1** ($CDCl_3$, 500 MHz).

WILEY-VCH

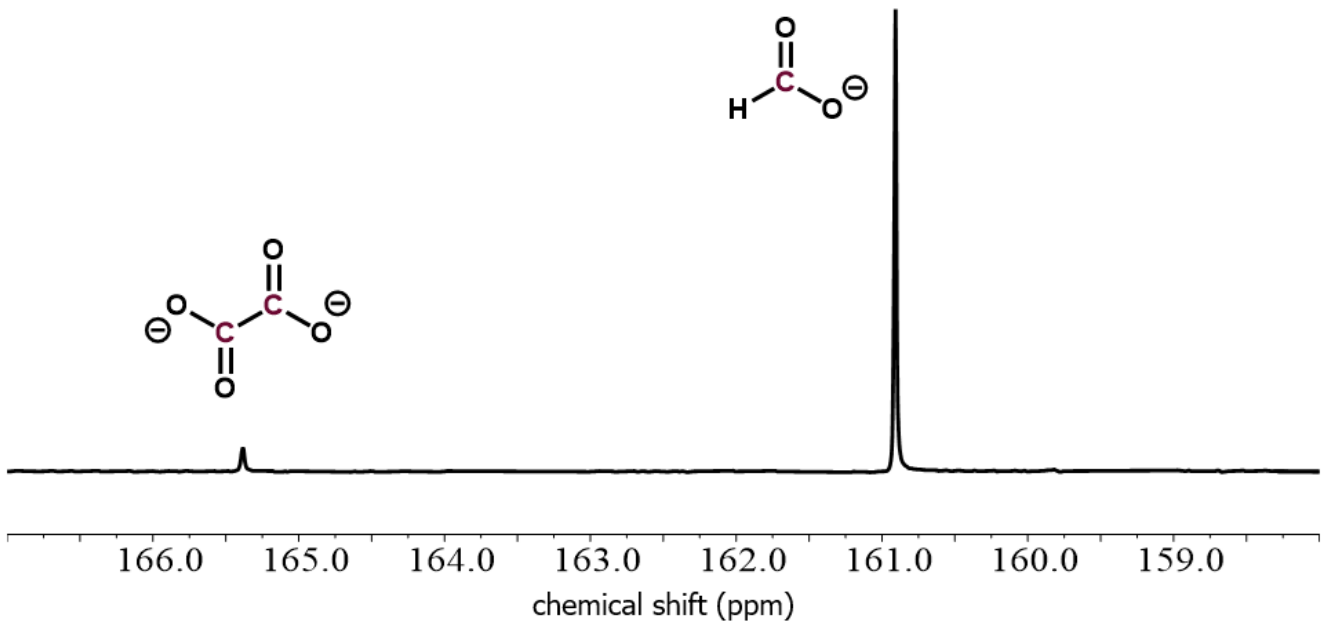

Figure S6. ^{13}C NMR of reaction mixture after 13 hours of CO_2 reduction by **Phen-COF** (D_2O, 500 MHz).

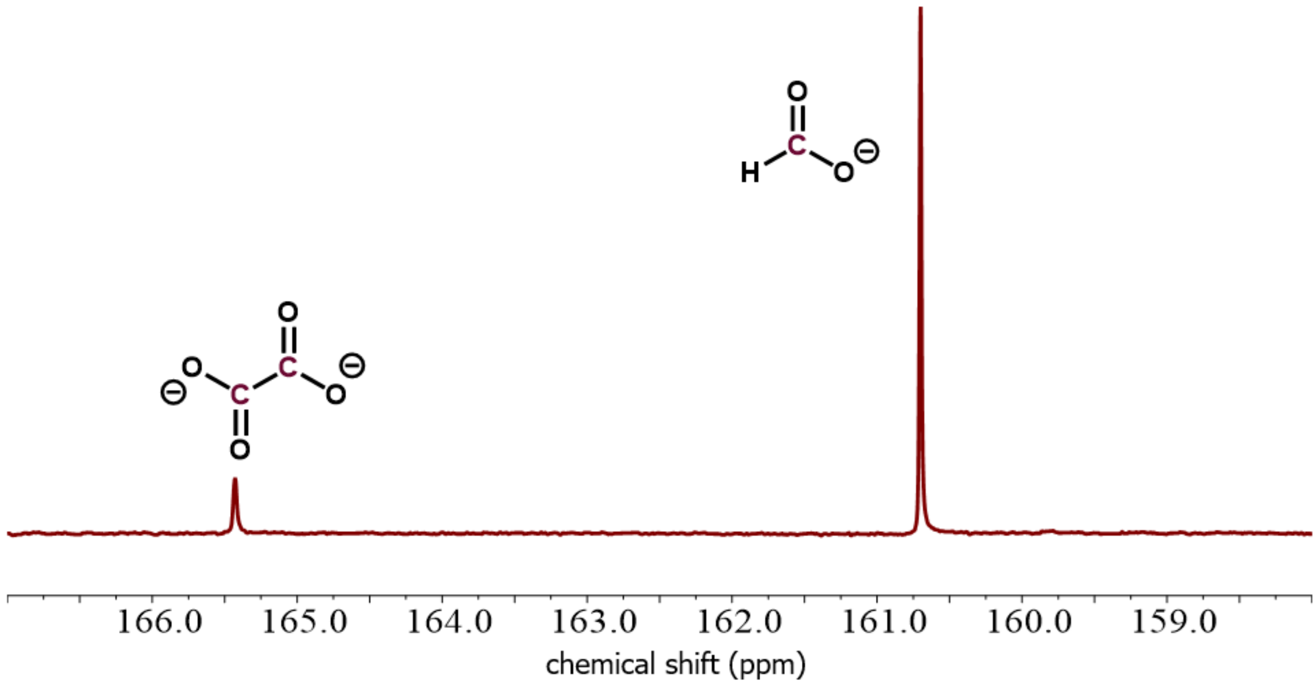

Figure S7. ^{13}C NMR of reaction mixture after 13 hours of CO_2 reduction by **1** (D_2O, 500 MHz).

III. FT-IR Spectra

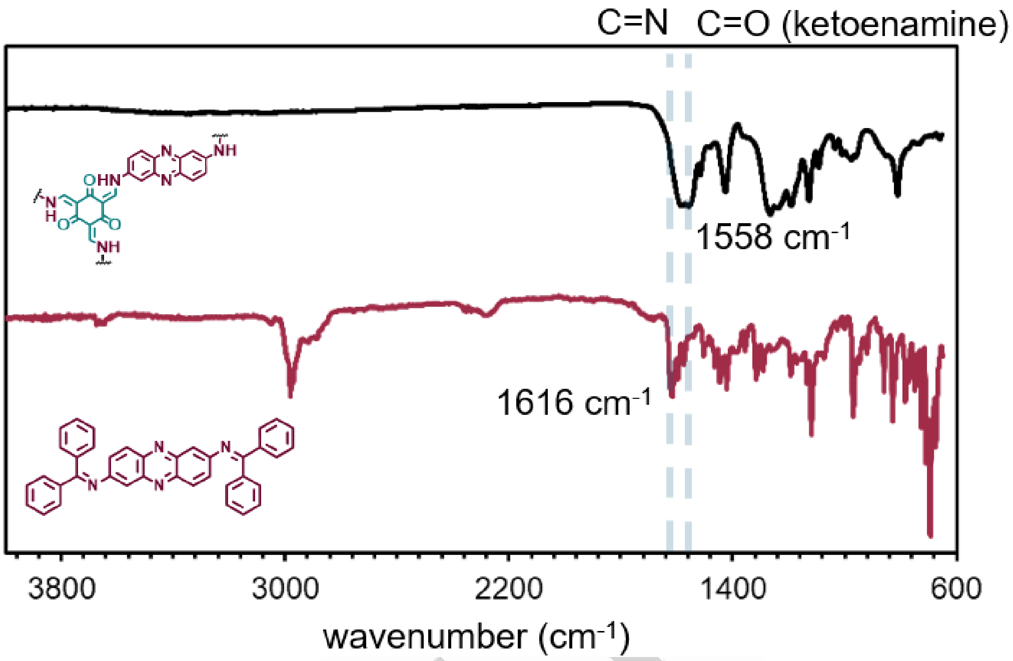

Figure S8. Fourier Transform IR spectra of **Phen-COF** (black) and **1** (maroon).

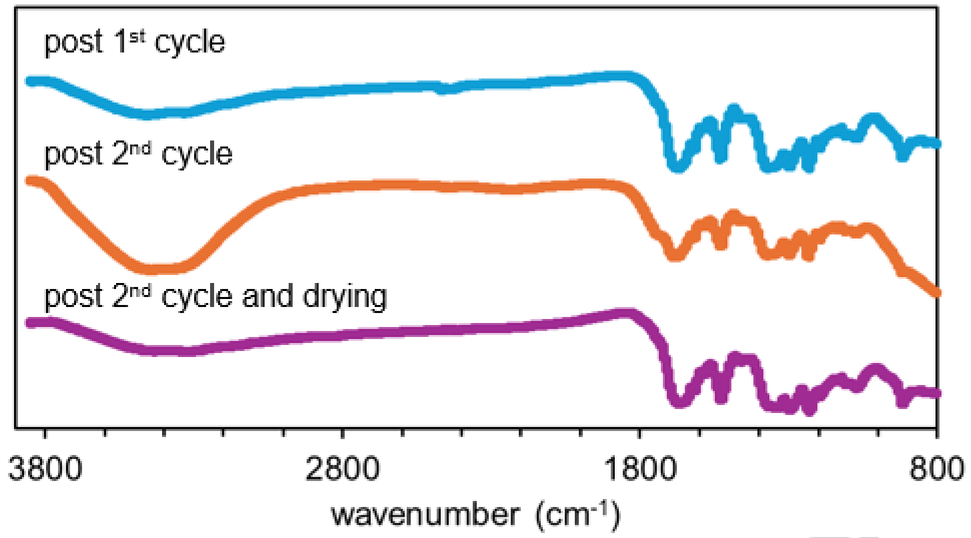

Figure S9. Fourier Transform IR spectra of **Phen-COF** after two catalytic cyclings and drying.

IV. X-Ray Photoelectron Spectroscopy

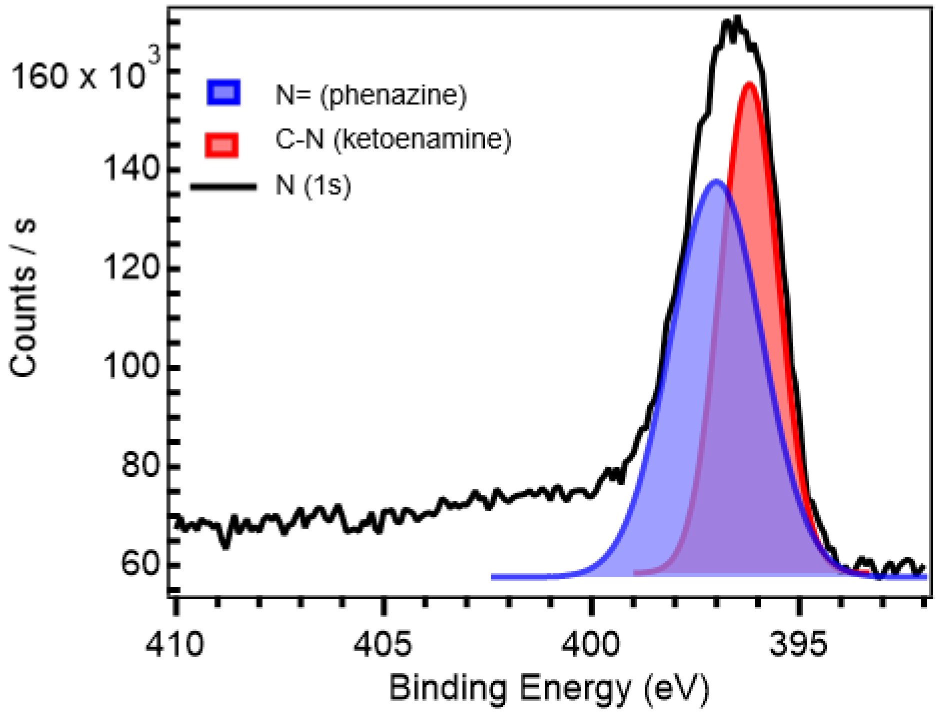

Figure S10. X-ray photoelectron spectrum of N_{1s} binding energy in **Phen-COF.**

WILEY-VCH

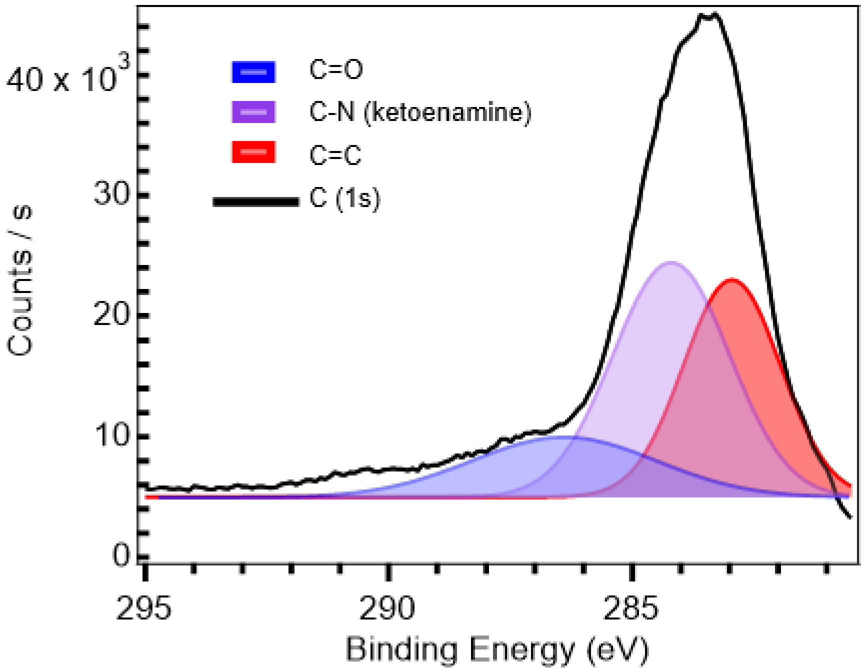

Figure S11. X-ray photoelectron spectrum of C_{1s} binding energy region in **Phen-COF.**

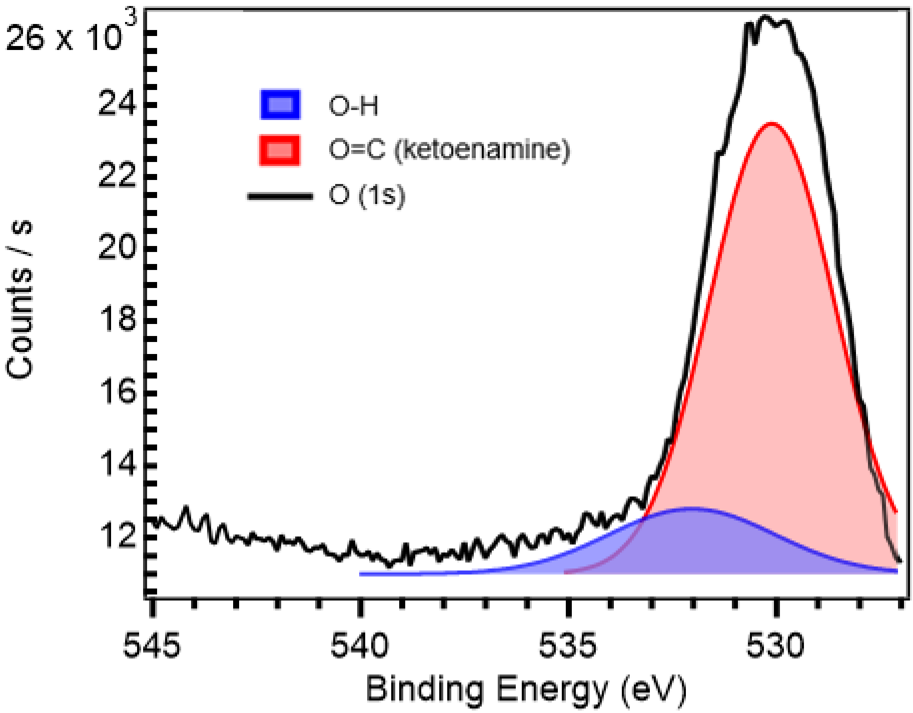

Figure S12. X-ray photoelectron spectrum of the O_{1s} binding energy region in **Phen-COF.**

V. Thermogravimetric Analysis

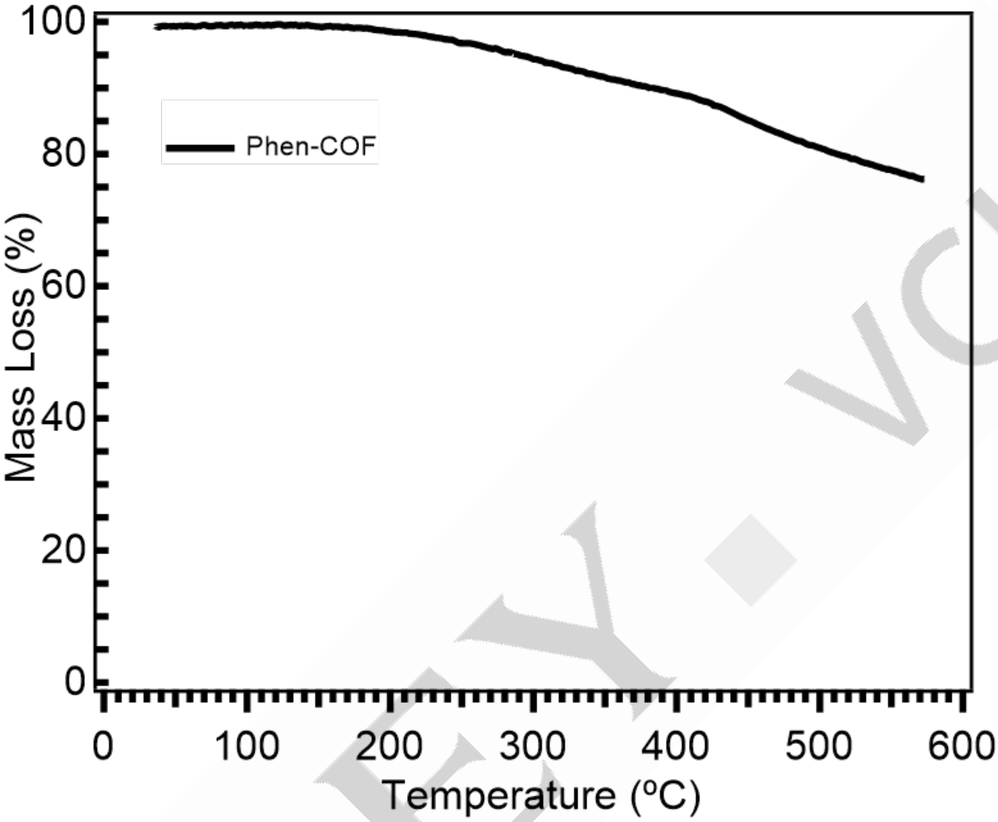

Figure S13. Thermogravimetric analysis of **Phen-COF**.

WILEY-VCH

VI. Simulation of X-ray Diffraction Patterns

The Accelrys Materials Studio[42] (version 5.0) program suite was used to simulate the powder diffraction pattern of **Phen-COF**. The initial structure was modeled using a hexagonal unit cell with a P6/m space group. The ***a*** cell parameter was estimated from the <100> Bragg peak observed in the PXRD data. The initial interlayer stacking, represented by the ***c*** cell parameter, was set to 3.4 Å, which is consistent with previously reported similar COF structures.[39,43] The structure was optimized using the Geometry Optimization routine including energy minimization with cell parameters optimization, using the parameters from the Universal Force Field.[44,45] The simulated PXRDs were calculated with the Reflex Plus module. The experimental data was subjected to a Pawley refinement where the peak positions and line shape parameters were refined using the Pseudo-Voigt peak shape position[42] until further refinements resulted in no additional changes.[44,45]

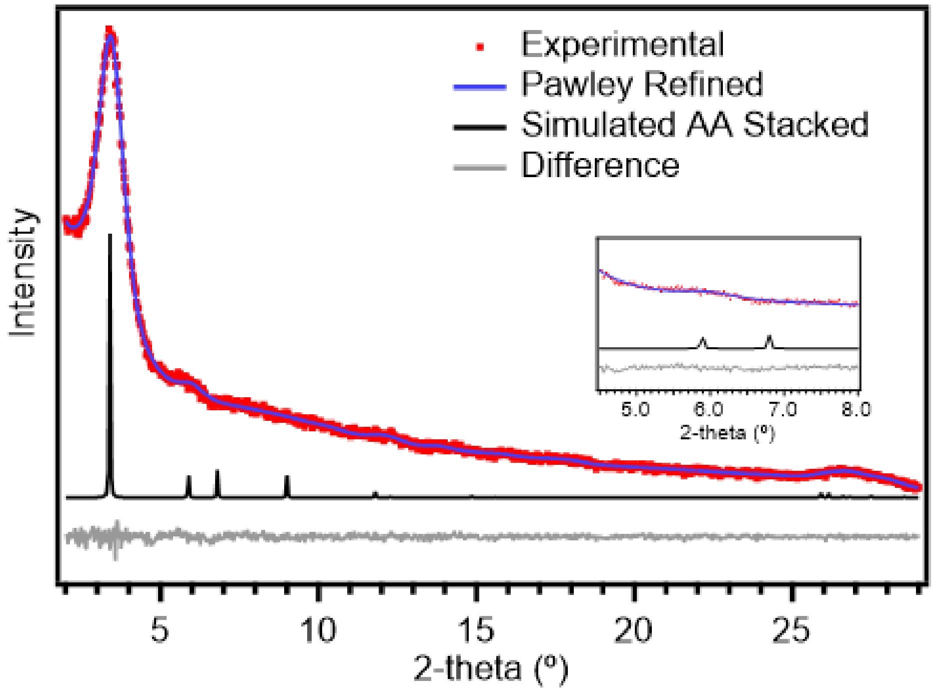

Figure S14. Phen-COF PXRD patterns for experimental (red), Pawley-refined (blue), and simulated average AA stacking (black).

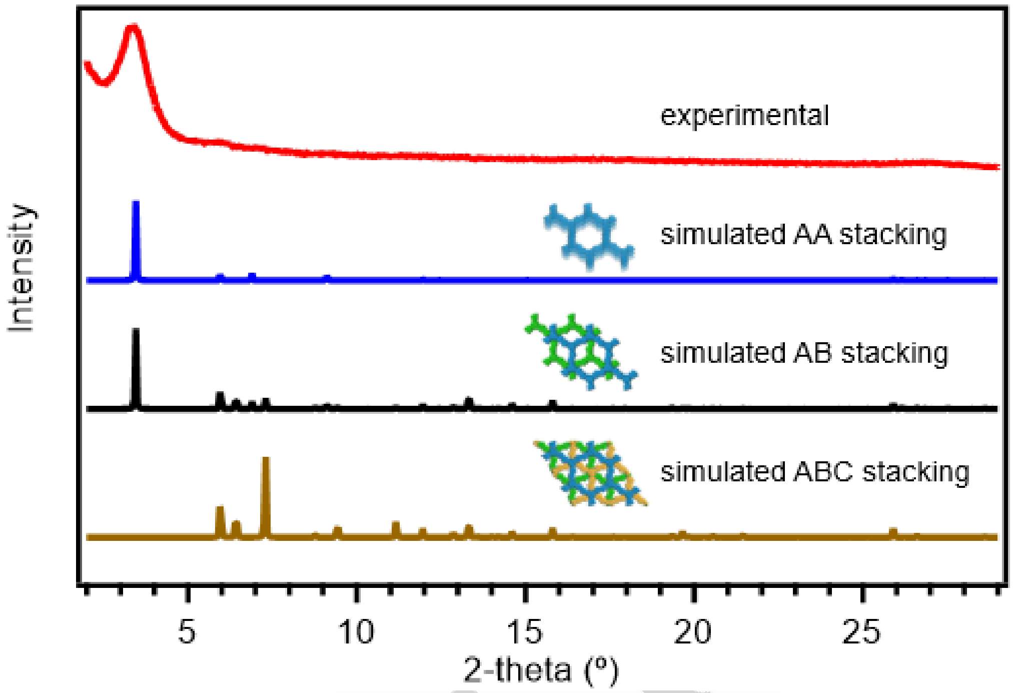

Figure S15. Phen-COF PXRD patterns for experimental (red), simulated average AA stacking (blue), simulated average AB stacking (black), and simulated ABC stacking (brown).

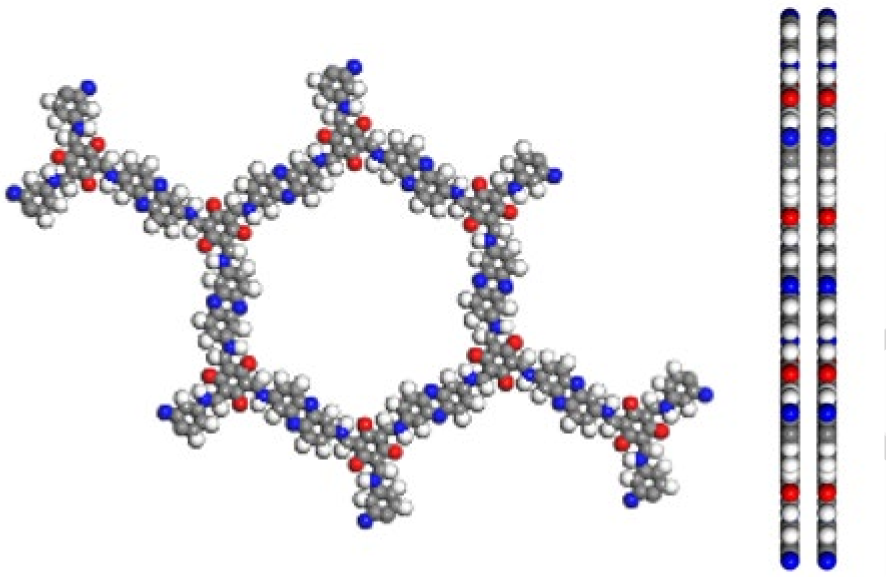

Figure S16. Geometry- and energy-minimized structure of AA-stacked **Phen-COF** built in Materials Studio.

WILEY-VCH

Table S1. Fractional atomic coordinates for unit cell of AA-stacked DAPH-TFP COF calculated using the Materials Studio modeling program. Symmetry space group = P6/m (175); Cell length: $a = b = 29.5940$; $c = 3.4366$.

Atom	*x*	*y*	*z*
C1	0.68138	0.38585	0.00000
C2	0.62848	0.34763	0.00000
C3	0.58823	0.35815	0.00000
N4	0.59612	0.40845	0.00000
C5	0.55506	0.41806	0.00000
C6	0.50177	0.37881	0.00000
N7	0.44674	0.46193	0.00000
C8	0.48147	0.4464	0.00000
C9	0.53491	0.48409	0.00000
C10	0.46459	0.39366	0.00000
C11	0.57065	0.46997	0.00000
O12	0.69584	0.43217	0.00000
H13	0.44764	0.67062	0.00000
H14	0.51227	0.66251	0.00000
H15	0.57435	0.6337	0.00000
H16	0.39074	0.5014	0.00000
H17	0.63189	0.44184	0.00000

WILEY-VCH

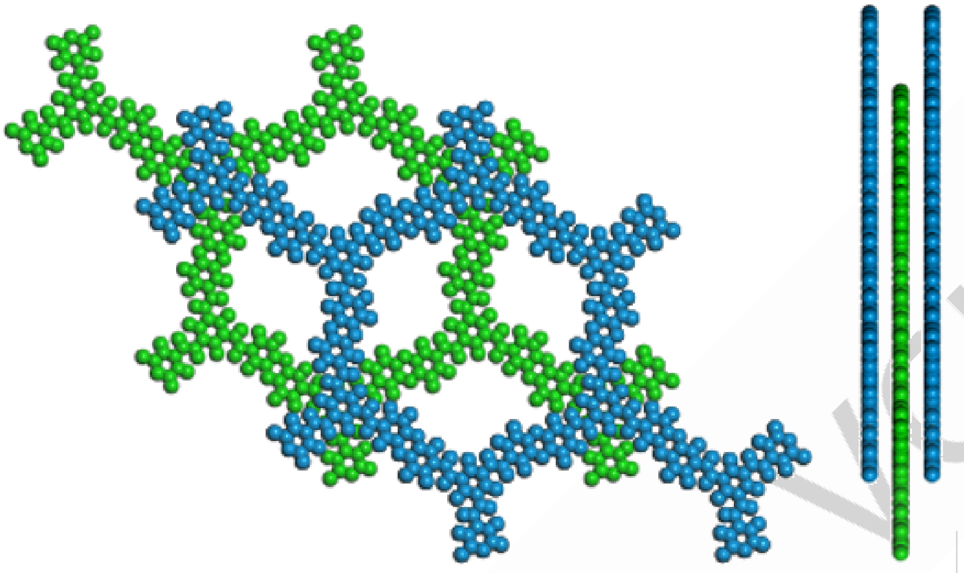

Figure S17. Geometry- and energy-minimized structure of AB-stacked **Phen-COF** built in Materials Studio.

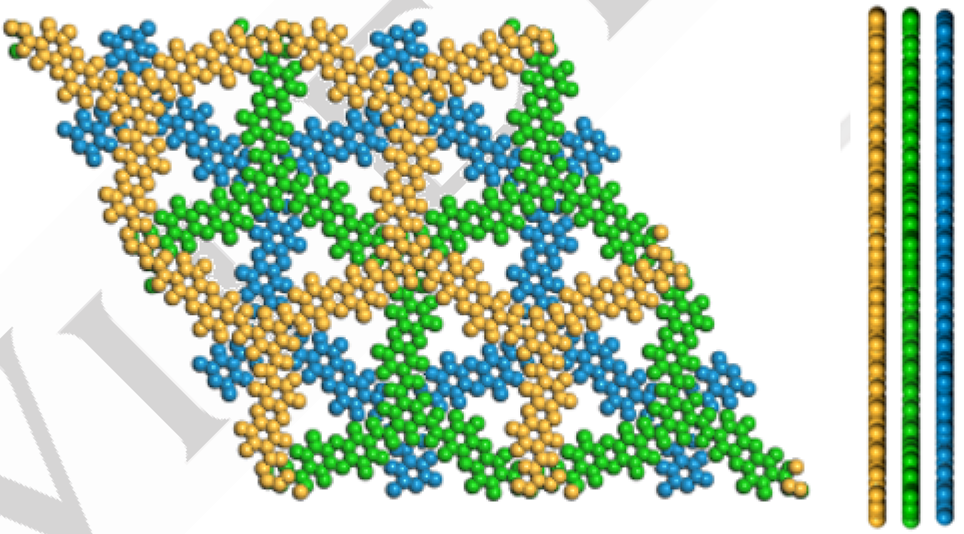

Figure S18. Geometry- and energy-minimized structure of ABC-stacked **Phen-COF** built in Materials Studio.

VII. Powder X-ray Diffraction Data

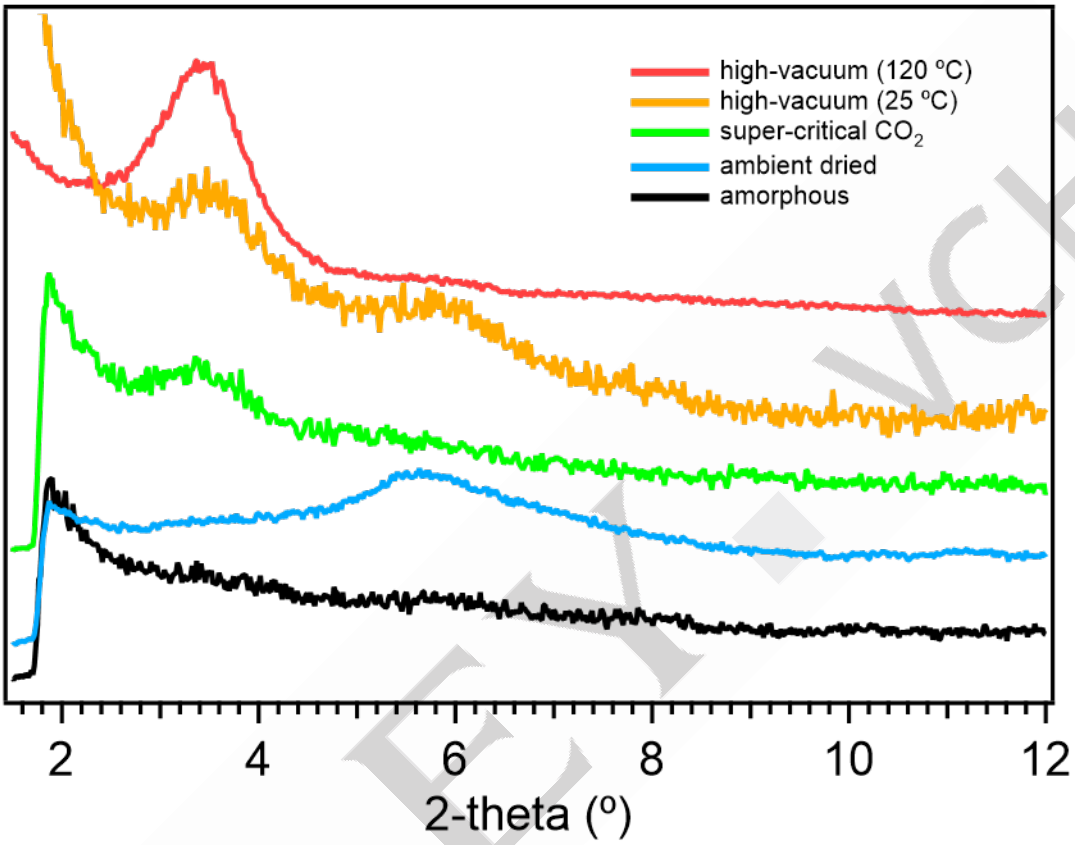

Figure S19. PXRD pattern of **Phen-COF** after post-synthetic solvent washing and drying by ambient conditions (blue), super-critical CO_2 (green), high-vacuum at room temperature (orange), and high-vacuum at 120 °C (red).

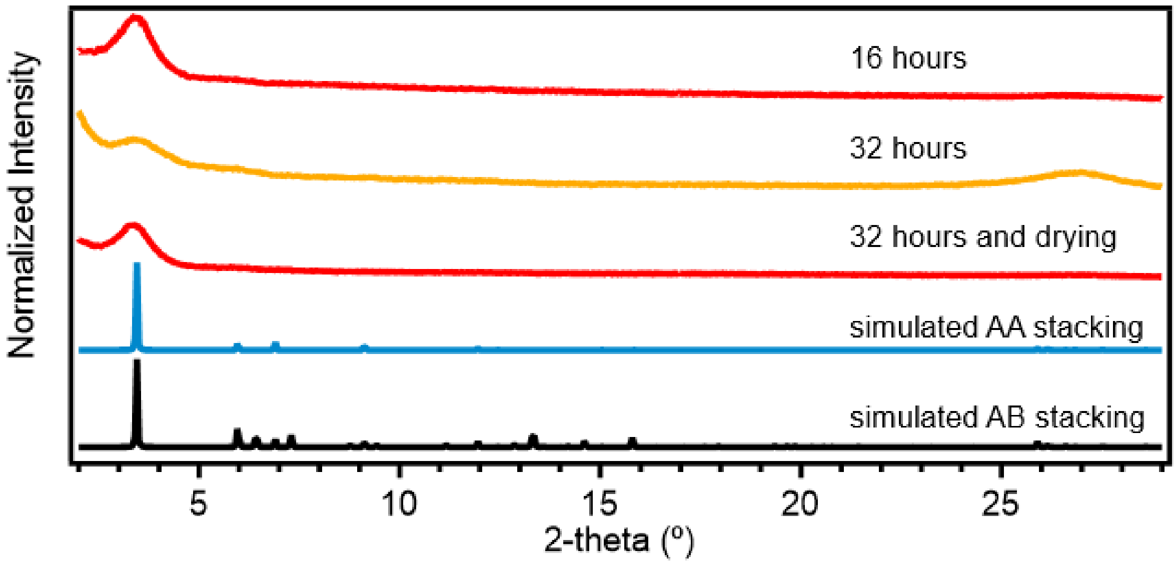

Figure S20. PXRD pattern of **Phen-COF** after one and two rounds of photocatalytic testing followed by drying by high-vacuum at 120 °C (red). Simulated PXRD patterns of average AA stacking (blue) and average AB stacking (black) are shown in comparison.

WILEY

VIII. Surface Area Measurements with BET insets

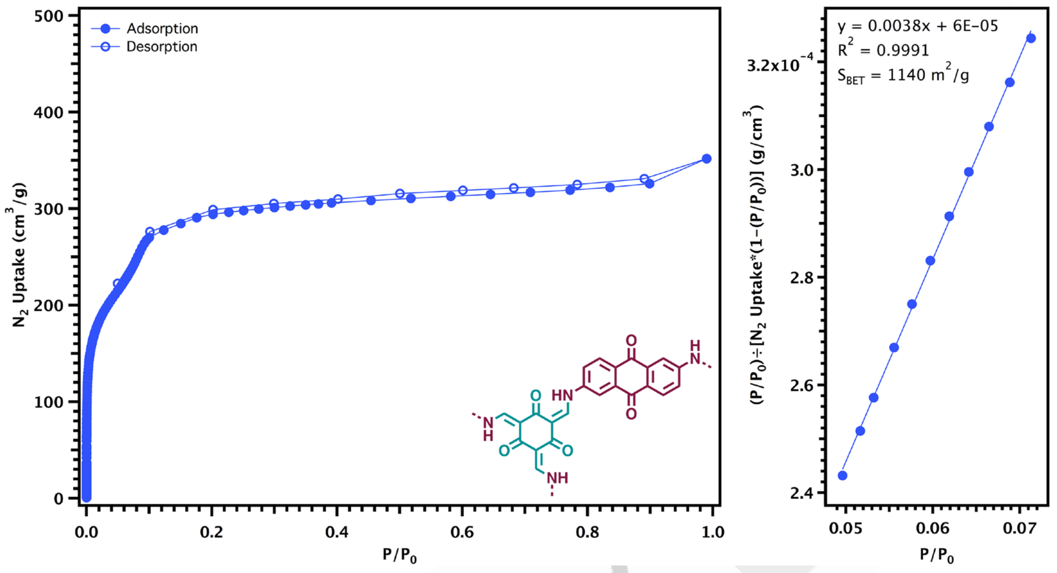

Figure S21. (Left) Adsorption Isotherm and (Right) BET surface area plot for **Phen-COF**.

IX. GCMS Methods and Data

GCMS Standardization. The custom glass photoreactor was sealed to the gas flow cell connected to separate lines for nitrogen, carbon dioxide, helium, and a dilute tank containing 0.5% carbon monoxide. The flow rates of different gases were controlled and monitored by a flow/pressure meter (Alicat). In each standardization injection, the GCMS trace was analyzed for ion current signal. Integrations of carbon (m/z = 12) from carbon monoxide were plotted against the concentration of its gas determined from the flow meter **(Figure S16).** Similar standard curves were prepared for oxygen (m/z = 16) and carbon dioxide (m/z = 44).

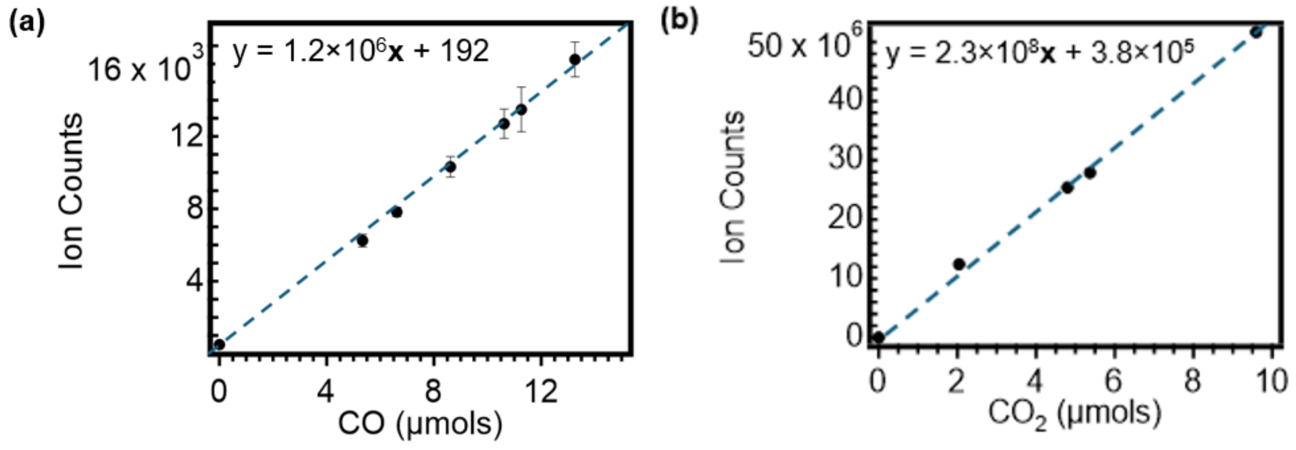

Figure S22. Standard curve for (a) CO and (b) CO_2 determined from ion counts versus concentration.

WILEY-VCH

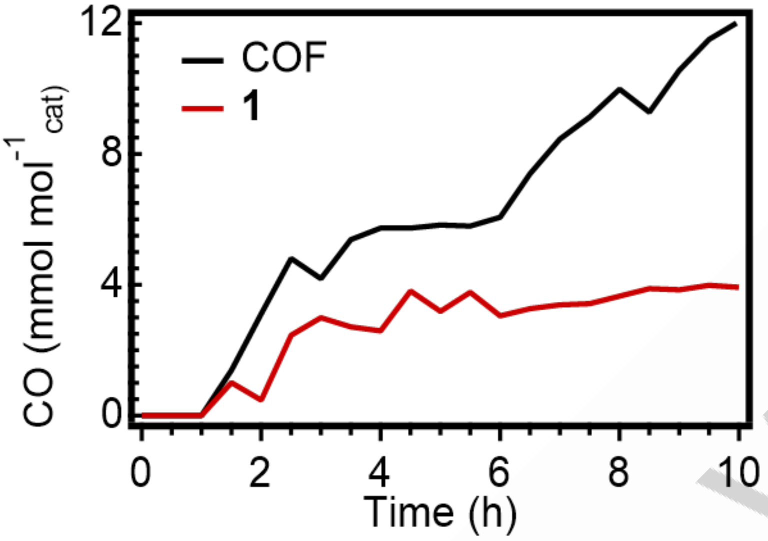

Figure S23. Traces of CO production over time for **Phen-COF** and **1** during irradiation at 467 nm (Kassel LED lamp). The flow rate was maintained at a pressure of 18.5 psi, and CO amounts were determined by *in operando* GCMS (m/z = 12 integrations).

WILEY-VCH

X. Photocatalytic Measurements

Phen-COF (13.3 mg, 444.5 g/mol) or compound 1 (10.6 mg, 540.67 g/mol) was sonicated in 2 mL of a solvent mixture consisting of H_2O/i-PrNEt/CH_3CN in a 7/2/1 ratio. For compound **1**, the resulting solution was pipetted directly into a custom photoreactor. In the case of the COF, after 5 minutes of sonication, an unstable slurry formed, which was pipetted into the photoreactor. The reactor was thoroughly degassed and backfilled with CO2. Illumination was provided by a Kassil Lamp (PR160L) with a maximum output of 52 W. During the irradiation, gas products were monitored using gas chromatography, and CO was quantified using an FID detector.

The percent conversion of CO_2 into products was determined by measuring the loss of CO_2 and the appearance of CO. In a typical experiment using 13.3 mg of **Phen-COF**, we determined that 1.87% of CO_2 is converted into CO, as 56.1 μmol of CO was produced as 3 mmol of CO_2 was consumed.

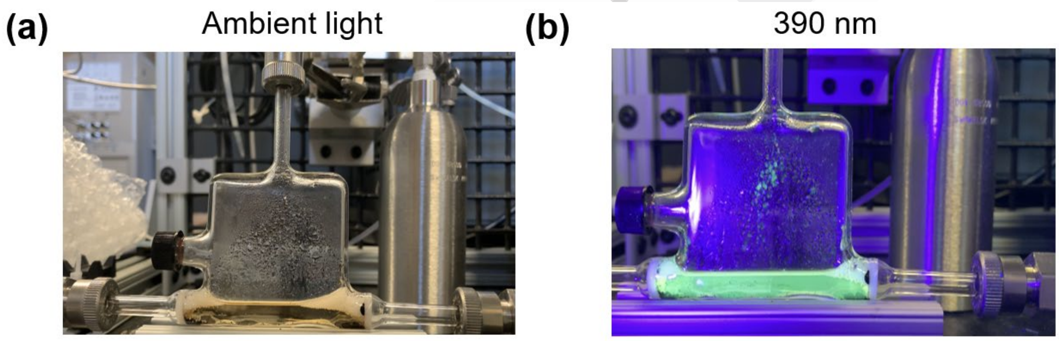

Figure S24. (a) Image of CO_2 flowing into photoreactor containing COF under ambient light and (b) under irradiated light at 390 nm.

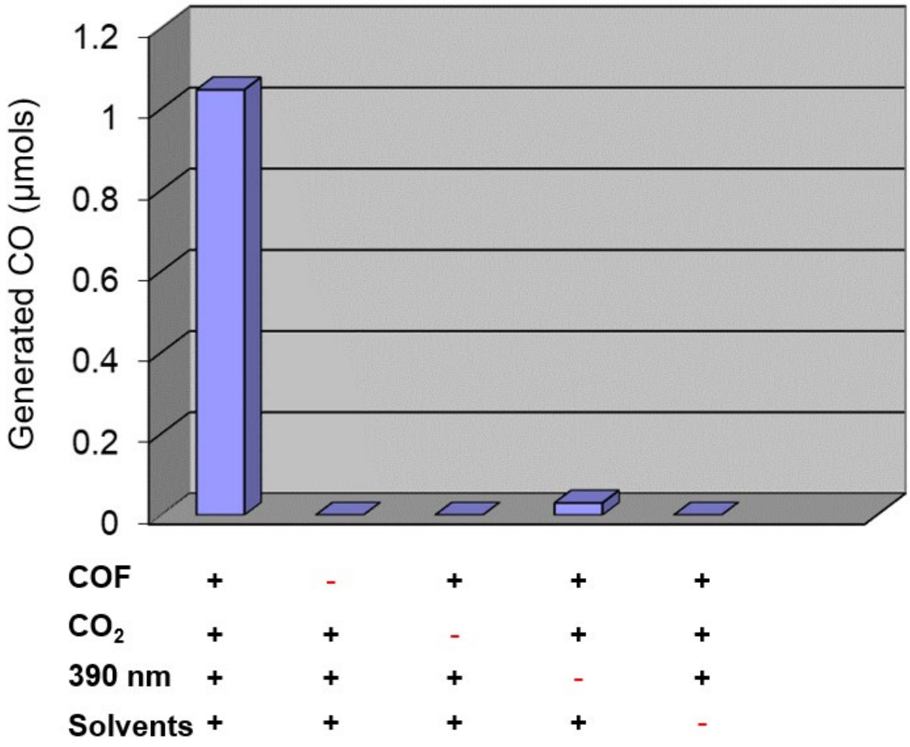

Figure S25. Control CO generation experiments for CO_2 reduction by **Phen-COF** in the absence of CO_2 starting material, irradiated light, and the presence of solvent/electron donor.

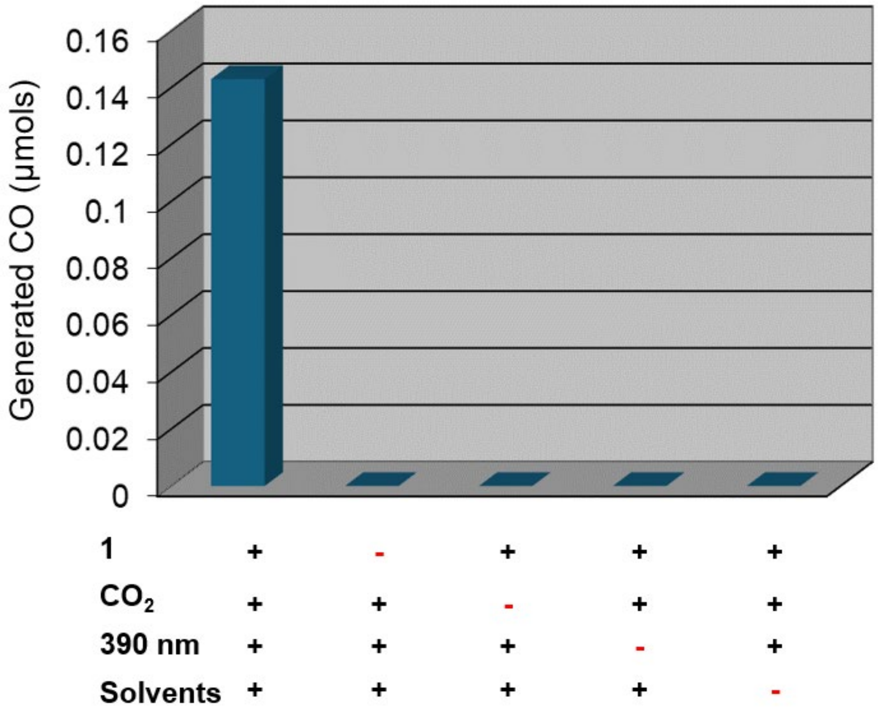

Figure S26. Control CO generation experiments for CO_2 reduction by **1** in the absence of CO_2 starting material, irradiated light, and the presence of solvent/electron donor.

WILEY-VCH

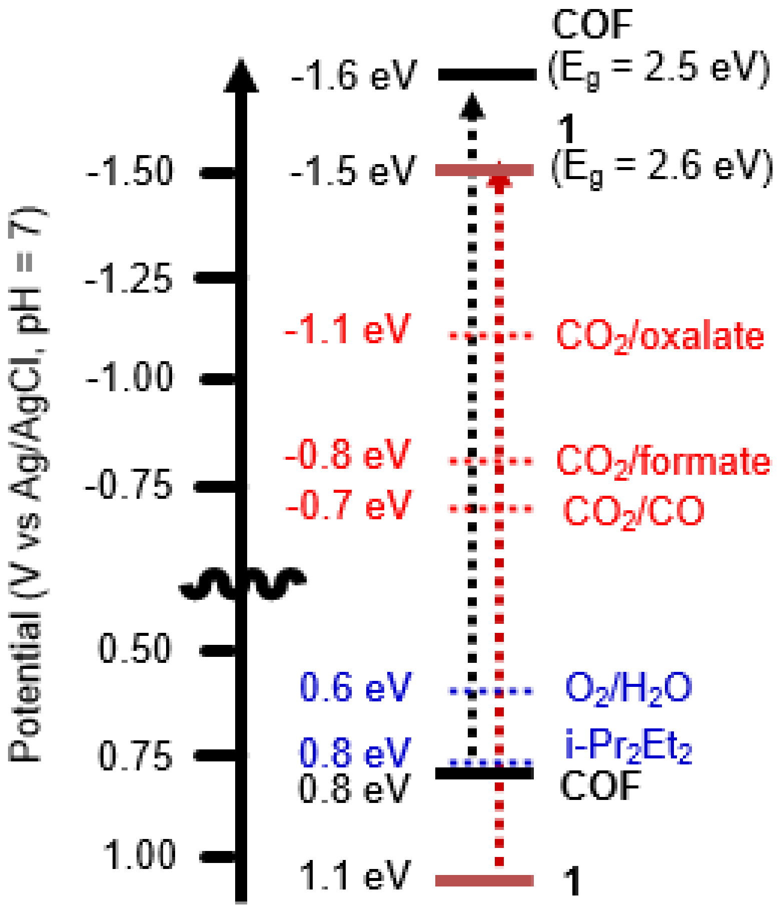

Figure S27. Graphical representation of relevant reduction and oxidation potentials of **Phen-COF** and **1** along with relevant products.

XI. Ion Chromatography Methods and Data

Ion Chromatography Standardization. Solutions containing both formate and oxalate were prepared in nanopure water (ranging from 5 to 200 μM). In each standardization injection, the ion chromatography trace was analyzed for conductivity. Integrations of the peaks were plotted against the prepared concentration.

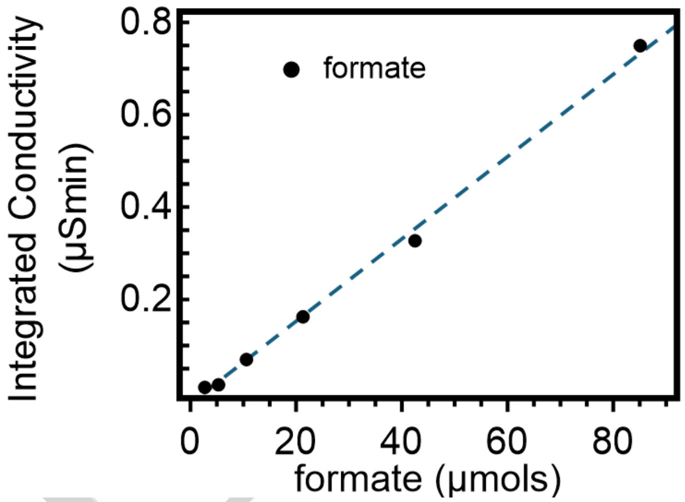

Figure S28. Standard curve for formate ion counts versus concentration.

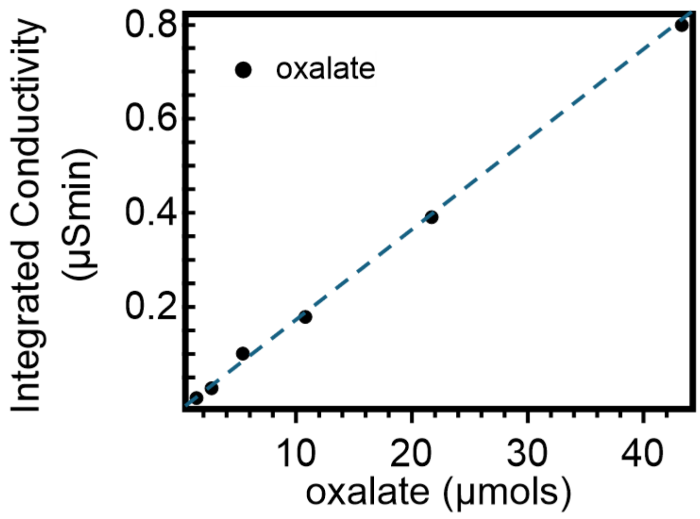

Figure S29. Standard curve for oxalate ion counts versus concentration.

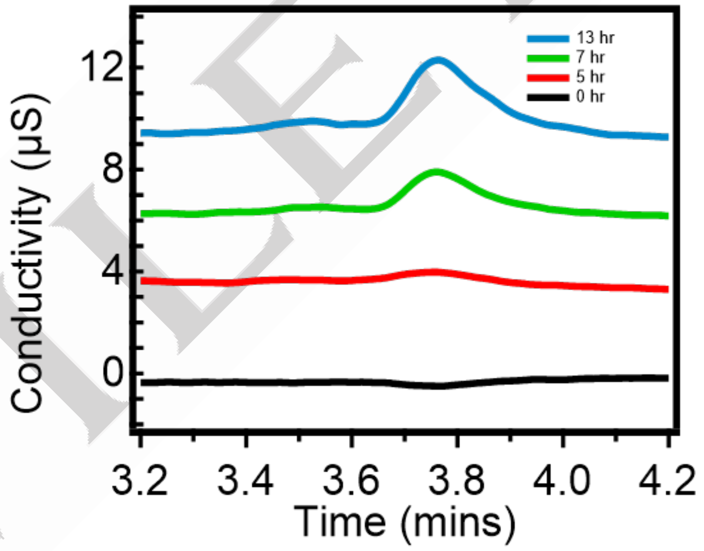

Figure S30. Time-interval acquired ion chromatograph traces of the reaction mixture after 13 hours of CO_2 reduction by **1,** zoomed in on formate retention time (irradiation at 390 nm).

WILEY-VCH

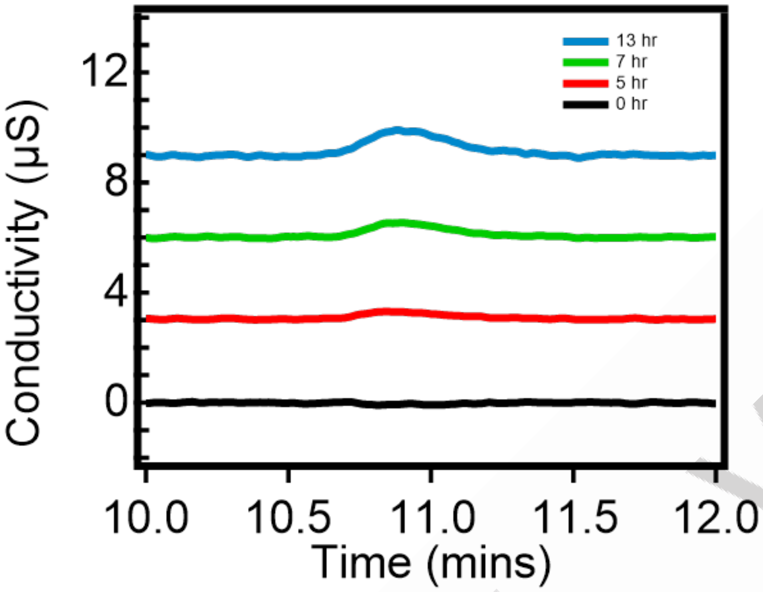

Figure S31. Time-interval acquired ion chromatograph traces of the reaction mixture after 13 hours of CO_2 reduction by **1,** zoomed in on oxalate retention time (irradiation at 390 nm).

WILEY-VCH

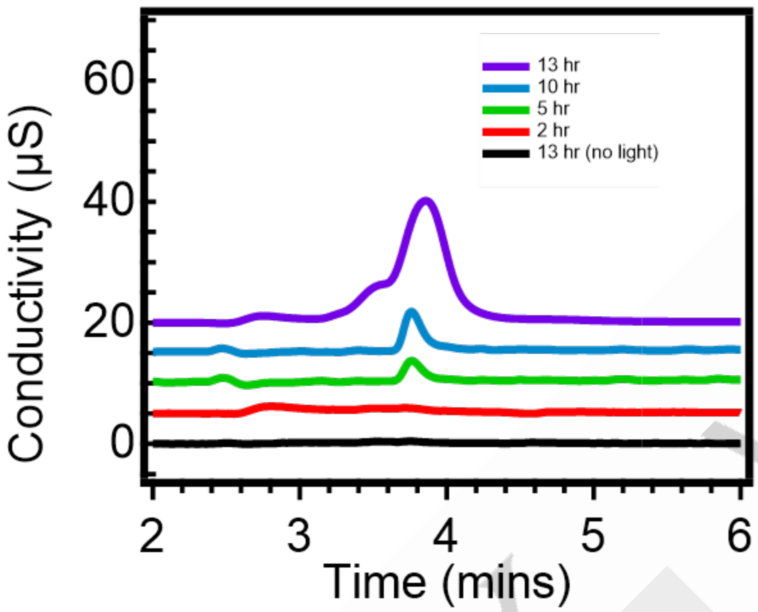

Figure S32. Time-interval acquired ion chromatograph traces of the reaction mixture after 13 hours of CO_2 reduction by **Phen-COF,** zoomed in on formate retention time.

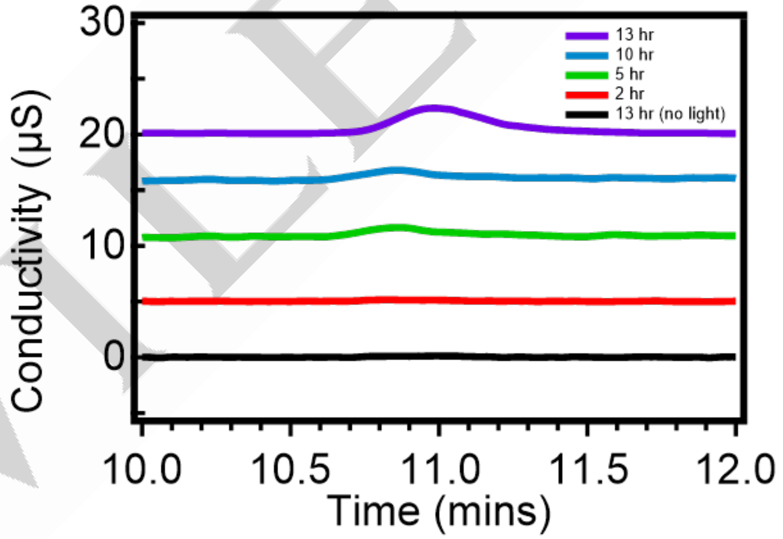

Figure S33. Time-interval acquired ion chromatograph traces of the reaction mixture after 13 hours of CO_2 reduction by **Phen-COF,** zoomed in on oxalate retention time.

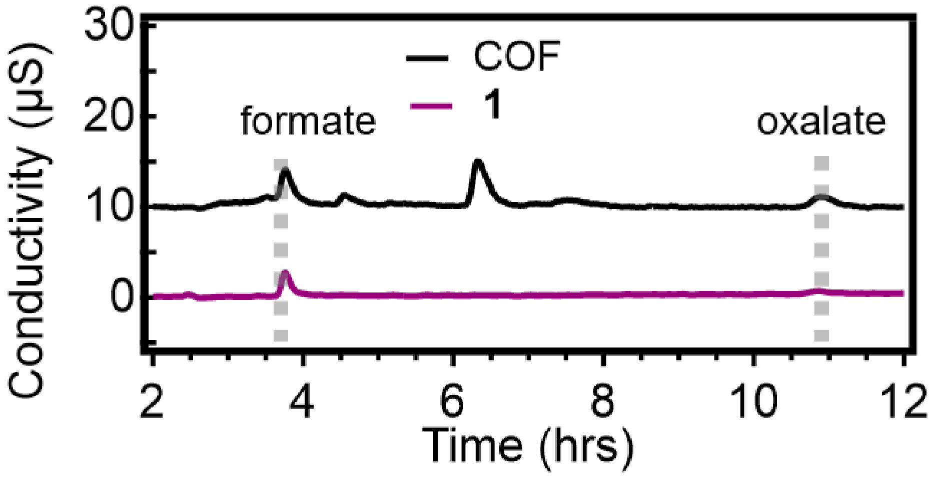

Figure S34. Time-interval acquired ion chromatograph traces of the reaction mixture after 10 hours of CO_2 reduction by **Phen-COF** and **1** after irradiation at 467 nm.

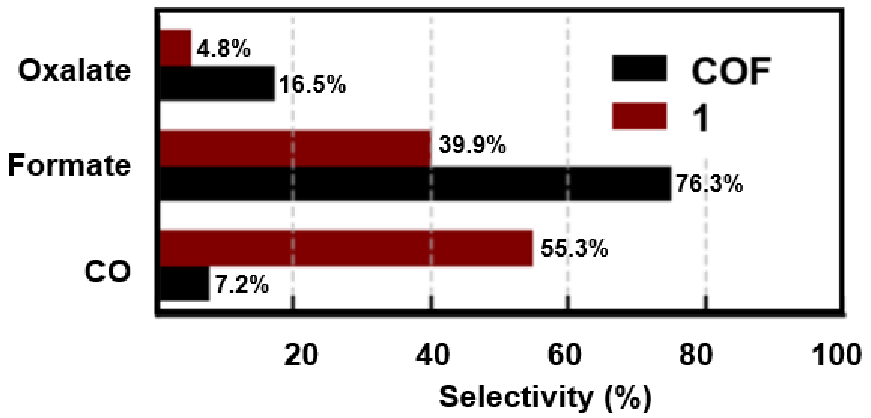

Figure S35. Product selectivity of **Phen-COF** and **1** for CO, formate, and oxalate production.

WILEY-VCH

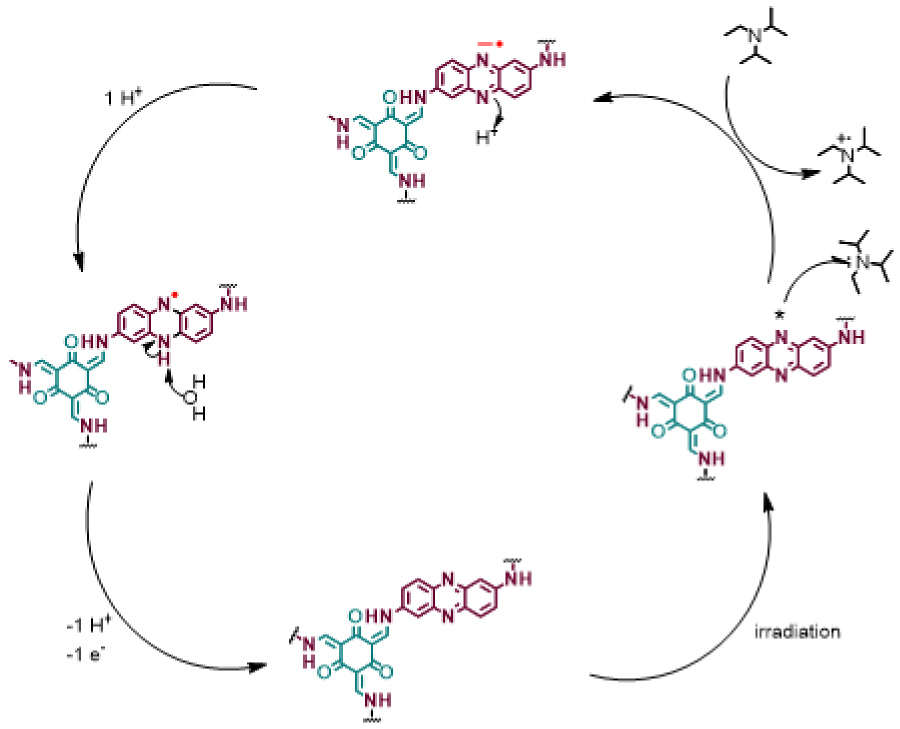

Figure S36. Proposed mechanism of catalyst regeneration in **Phen-COF**.

WILEY-VCH

XII. Comparison to Other COF Photocatalysts

Table S2. Photocatalytic evolution of products from metal-free COF-containing systems.

Material	Product evolution (µmol/g/h)	Product	Reference
ACOF-1	0.36	CH_3OH	[33]
N3-COF	0.57	CH_3OH	[33]
TpBb-COF	52.8	CO	[32]
LZU1-COF	25	CO (CH_4)	[50]
QL-COF	156	CO (CH_4)	[50]
2D CN-COF	85.8	CO	[64]
2D CN-COF	2.37	CH_4;	[64]
g-C3N4 (NH)-COF	123	CO	[65]
TpPa-1 on reduced graphene oxide	199	CO	[66]
**Phen-COF**	3.1	CO	this work
2,7-(diaminobenzophenone) phenazine (**1**)	1.1	CO	this work

WILEY-VCH

Table S3. Photocatalytic evolution of products from metal-free photocatalysts.

Material	Product evolution (μmol/g/h)	Product	Reference
graphene oxide	0.172	CH_3OH	[24]
conjugated polymer (CPs-B)	267	CO	[27]
conjugated polymer (CPs-Th)	667	CO	[27]
conjugated polymer (CPs-BT)	1213	CO	[27]
g-C_3N_4 ultrathin nanosheets	1.9	CO	[69]
Helical g-C_3N_4 nanorods	89.0	CO	[70]
Porous nitrogen-rich g-C_3N_4 nanotubes	103.6	CO	[71]
**Phen-COF**	3.1	CO	this work
2,7-(diaminobenzophenone) phenazine (**1**)	1.1	CO	this work

WILEY-VCH
